# Systematic Identification of Cell-Cell Communication Networks in the Developing Brain

**DOI:** 10.1016/j.isci.2019.10.026

**Published:** 2019-10-17

**Authors:** Bilal N. Sheikh, Olga Bondareva, Sukanya Guhathakurta, Tsz Hong Tsang, Katarzyna Sikora, Nadim Aizarani, Sagar, Herbert Holz, Dominic Grün, Lutz Hein, Asifa Akhtar

**Affiliations:** 1 Max Planck Institute of Immunobiology and Epigenetics, Stuebeweg 51, Freiburg 79108, Germany; 2 Institute of Experimental and Clinical Pharmacology and Toxicology, University of Freiburg, Albertstr. 25, Freiburg 79104, Germany; 3 Faculty of Biology, Albert Ludwig University of Freiburg, Freiburg 79104, Germany

**Keywords:** Neuroscience, Developmental Neuroscience, Systems Neuroscience, Techniques in Neuroscience

## Abstract

Since the generation of cell-type specific knockout models, the importance of inter-cellular communication between neural, vascular, and microglial cells during neural development has been increasingly appreciated. However, the extent of communication between these major cell populations remains to be systematically mapped. Here, we describe EMBRACE (embryonic brain cell extraction using FACS), a method to simultaneously isolate neural, mural, endothelial, and microglial cells to more than 94% purity in ∼4 h. Utilizing EMBRACE we isolate, transcriptionally analyze, and build a cell-cell communication map of the developing mouse brain. We identify 1,710 unique ligand-receptor interactions between neural, endothelial, mural, and microglial cells *in silico* and experimentally confirm the APOE-LDLR, APOE-LRP1, VTN-KDR, and LAMA4-ITGB1 interactions in the E14.5 brain. We provide our data via the searchable “Brain interactome explorer”, available at https://mpi-ie.shinyapps.io/braininteractomeexplorer/. Together, this study provides a comprehensive map that reveals the richness of communication within the developing brain.

## Highlights

• Isolation of embryonic neural, mural, endothelial, and microglial cells to >94% purity
• Transcriptome analyses of neural, vascular, and microglial cells from E14.5 brain
• Generation of inter-cellular communication network with 1,710 unique interactions
• Established “Brain interactome explorer,” a searchable cell communication database

## Graphical Abstract

**Figure:**
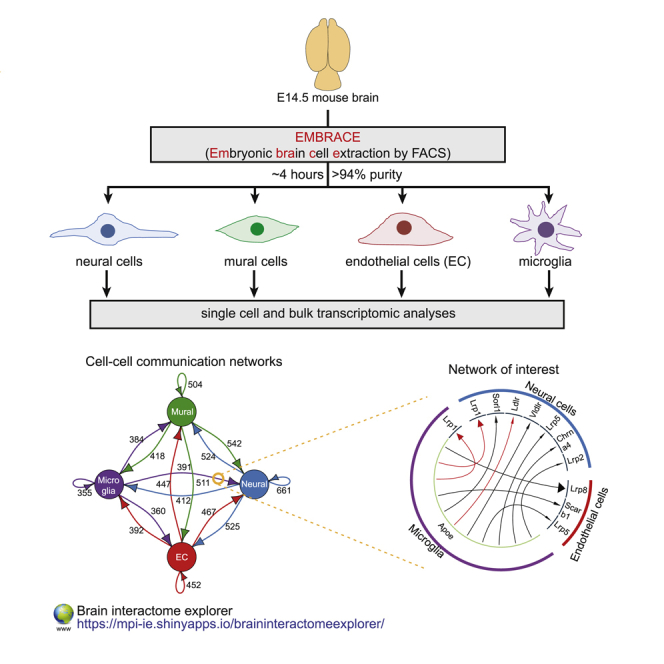

## Introduction

Embryonic development is a highly reproducible process that requires extensive communication between cells. Inter-cellular communication is particularly evident during the development and maturation of the mammalian brain. During embryonic development, human and murine brains consist primarily of neural stem cells that give rise to progenitors, which migrate into the developing cortex and differentiate into neurons (Jiang and Nardelli, 2016). In contrast, astrocytes and oligodendrocytes start to develop around the time of birth and are largely absent during prenatal development. In addition to the neural lineage, microglia, the resident immune cells of the central nervous system, as well as vascular endothelial cells and pericytes are also present in the developing brain (Alliot et al., 1999, Daneman et al., 2010b, Vasudevan et al., 2008). The presence and functionality of vascular cells and microglia is critical for proper neural development, and dysregulation of these cells results in severe neural disorders (Daneman et al., 2010b, Matcovitch-Natan et al., 2016, Mathys et al., 2017, Sengillo et al., 2013, Vasudevan et al., 2008). Indeed, neural cells not only communicate to impart particular cell fates upon each other (Barnabe-Heider et al., 2005, Yuzwa et al., 2016), but also communicate with developing vascular cells and microglia to guide their development (Haigh et al., 2003, Ma et al., 2017, Nikolakopoulou et al., 2013, Sellner et al., 2016). For instance, VEGF-A released by neural progenitor cells is detected by endothelial cells and is critical for proper angiogenesis and vascularization of the developing brain (Haigh et al., 2003). Similarly, endothelial cells are able to modulate the behavior of neural stem and progenitor cells (Crouch et al., 2015), as well as recruit vascular pericytes to ensure proper establishment of the blood-brain barrier (Hellstrom et al., 1999, Winkler et al., 2010). Simultaneously, pericytes provide differentiation signals for endothelial cells and modulate their function (Daneman et al., 2010b). Furthermore, microglia begin to enter the developing brain around E9 (Alliot et al., 1999, Stremmel et al., 2018) and modulate aspects of neural differentiation and synaptic structure (Nikolakopoulou et al., 2013, Paolicelli et al., 2011, Zhan et al., 2014). Despite the accumulation of evidence that interactions between neural cells, microglia, and vascular cells are critical for proper brain development, the identity of the molecules that mediate these inter-cellular interactions remains to be systematically mapped.

Fluorescence-activated cell sorting (FACS) is commonly used for the isolation and profiling of neural vascular cells and microglia. Microglia can be enriched using antibodies against CD11b and CD45 (Bennett et al., 2016, Datta et al., 2018, Mathys et al., 2017), whereas isolation of the mural cell population, which spans both pericytes and smooth muscle cells, typically relies on transgenic mice expressing fluorescent proteins under the *Pdgfrb* and *Cspg4* promoters (He et al., 2016, Vanlandewijck et al., 2018). Similarly, studies have utilized transgenic approaches such as *Tie2-GFP* (Daneman et al., 2010a, Zhang et al., 2014) and *Cldn5-GFP* (Vanlandewijck et al., 2018) animals for the isolation of endothelial cells. Given the time-consuming nature of transgenic animal production and crossing to mouse models of interest, researchers have been attempting to establish antibody-based methods for the isolation of vascular cells. Antibodies against CD13 (Crouch and Doetsch, 2018) and PDGFRβ (Epshtein et al., 2017) have recently been tested for the isolation of mural cells, whereas the use of antibodies against CD31 (PECAM1) is becoming more widespread for the isolation of endothelial cells (Crouch and Doetsch, 2018, Czupalla et al., 2018, Fan et al., 2014, Wang et al., 2019). The specificity of these markers has been confirmed using immunohistochemistry. However, the accuracy or purity of cell populations obtained from antibody-based FACS methods is yet to be quantifiably tested. Furthermore, given the importance of inter-cellular communication within the brain, a reliable and efficient method is still required to simultaneously isolate neural, vascular, and microglial cells to map changes in inter-cellular networks in genetically modified model systems.

In the current study, we describe EMBRACE (embryonic brain cell extraction using FACS), a method that allows for the simultaneous and rapid isolation of neural, mural, endothelial, and microglial cells from the embryonic brain. The combinations of cell-type specific markers utilized in EMBRACE permit it to achieve 94%–100% purity for each of the cell populations, which we validate through single cell RNA sequencing (scRNA-seq) analyses. To capture lowly expressed genes and to obtain better transcriptional resolution for in-depth analyses, we additionally perform low-input bulk RNA-seq on cell populations isolated by EMBRACE. Utilizing this transcriptomic data, we build a cell-cell communication network that reveals the richness and extent of communication within the developing brain.

## Results

### Sorting Strategy for the Isolation of Neural, Microglial, and Vascular Cells

In the current study, we set out to establish a protocol for the simultaneous isolation of neural, mural, endothelial, and microglial cells and systematically map interactions between these four cell types. We chose to focus our efforts on the E14.5 mouse brain for these analyses. The neural population in the E14.5 embryo consists primarily of neural stem and progenitors cells as well as migrating neurons (Jiang and Nardelli, 2016). Thus, cell dissociation methods are unlikely to cause excessive cell death as is common with mature neuronal populations, which possess extensive neurites. Furthermore, microglial seeding of the brain begins around E9 and is completed by E14.5 (Stremmel et al., 2018), suggesting that microglia would already be present and likely interacting with their native neural environment in the E14.5 brain. Neural vascularization and angiogenesis are also evident at E14.5 with the presence of maturing endothelial cells, active migration of tip cells, as well as recruitment and differentiation of mural cells (Tata et al., 2015). In fact, blood-brain barrier (BBB) maturation is completed around E15.5, suggesting that analyses at E14.5 are likely to reveal key factors required for BBB maturation.

To identify the most efficient method to dissociate E14.5 embryonic brains into a single cell suspension, we tested a number of enzymatic and non-enzymatic methods. We identified the combination of Liberase and DNase I as the most reliable method that gave the best cell viability (67.8%, Table S1). Therefore, we employed the combination of Liberase and DNase I for brain dissociation in all subsequent experiments.

**Figure 1:**
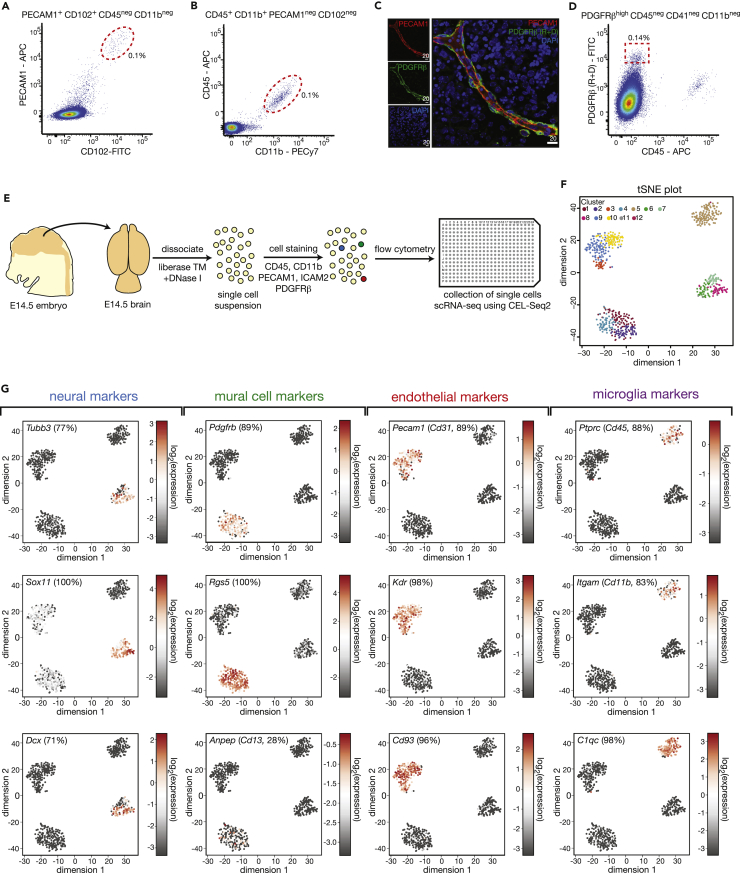
Identification of Cell Surface Markers for the Isolation of Neural, Microglial, and Vascular Cells (A) FACS plot showing enrichment of endothelial cells using PECAM1 and CD102 (ICAM2). (B) Exemplary FACS plot depicting the FACS strategy for the enrichment of microglia using CD11b and CD45. (C) Immunofluorescence image showing a blood vessel in the E14.5 brain stained for the mural cell marker PDGFRβ (green) and endothelial marker PECAM1 (red). The PDGFRβ antibody (from R+D) showed high specificity as evident by the strong peri-vascular staining. This is in contrast to other tested PDGFRβ and CSPG4 antibodies that showed unspecific staining (Figures S1H–S1L). Scale bars are provided in micrometers. (D) Sorting strategy for PDGFRβ-positive mural cells. (E) Schematic representation of scRNA-seq procedure used to test the specificity of the neural, microglial, and vascular cell isolation procedure. E14.5 mouse brains were dissociated using Liberase and DNase I, and the single cell suspension stained for selected cell surface markers followed by FACS of single cells into 384-well plates. Libraries for sequencing were prepared using the mCEL-Seq2 protocol. Cells were isolated in equal numbers from three independent wild-type E14.5 brains. (F) The t-distributed stochastic neighbor embedding (tSNE) plot generated with the RaceID3 package (Herman et al., 2018) of the 625 cells that were isolated using the selected cell surface markers and passed the filtering criteria (>1,500 unique transcripts). Twelve distinct clusters and four major cell populations were identified by unsupervised clustering. (G) Expression of neural, mural, endothelial, and microglial cell-type-specific genes. Each of the cell types was confined to one of the major clusters. Note the low levels of *Cd13* expression in the mural cell population. The numbers in parenthesis represent the proportion of cells in the respective cluster where marker expression was detectable. See also Figures S1 and S2.

To isolate the rare mural, endothelial, and microglial cell populations by FACS, we searched for cell surface proteins that are enriched in each of the cell types and screened for specific antibodies against these markers. We identified antibodies against PECAM1 (CD31) and CD102 that faithfully co-stained endothelial cells, as well as CD11b and CD45 antibodies that co-stained a microglial population (Figures 1A and 1B). We next searched for strongly expressed cell surface markers specific for the mural cell population. Utilizing a recently published single cell RNA sequencing dataset from the adult brain (Vanlandewijck et al., 2018), we chose to focus on *Pdgfrb* and *Cspg4* as they are both cell surface proteins and strongly expressed in all mural cells (Figures S1A–S1D). We screened antibodies against PDGFRβ and CSPG4 through immunofluorescence and FACS analyses and were able to identify a highly specific PDGFRβ antibody that we used in subsequent experiments (Figures 1C, 1D, and S1E–S1L).

**Figure 2:**
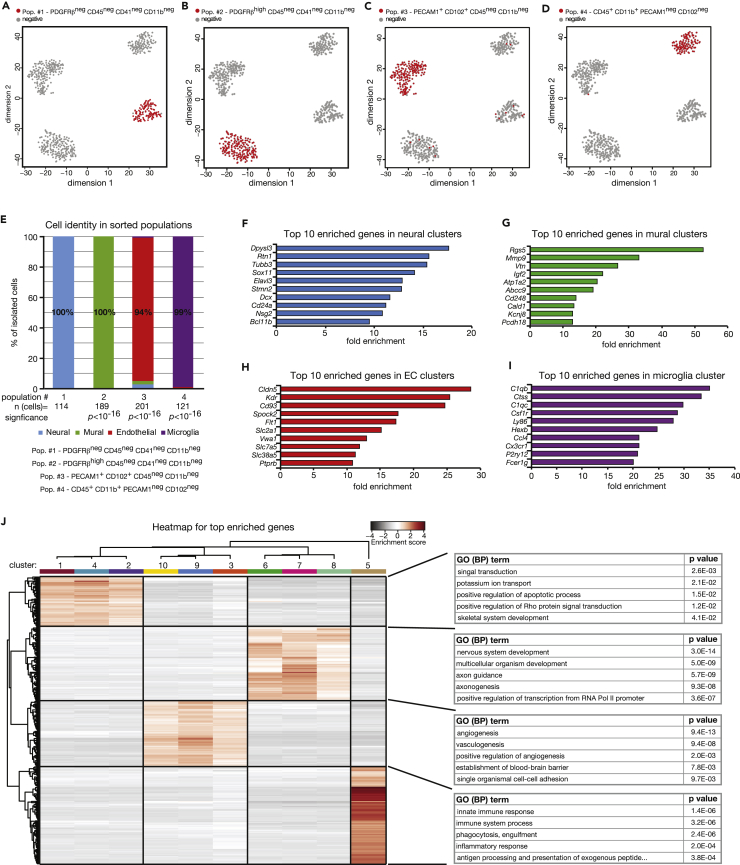
High Enrichment of Neural, Mural, Endothelial, and Microglial Cells Using EMBRACE (A–D) tSNE maps showing the clustering of cells sorted based on markers for (A) neural cells, (B) mural cells, (C) endothelial cells, and (D) microglia. (E) Identity of cells in each of the four EMBRACE-sorted populations based on the comparison of cell surface markers and cell identity, as specified by unsupervised hierarchical clustering of scRNA-seq data. (F–I) Top 10 genes enriched in (F) neural, (G) mural, (H) endothelial, and (I) microglia clusters. A more comprehensive list of genes enriched in each of the cell populations can be found in Tables S3–S6. (J) Heatmap depicting mean expression per cluster of top enriched genes in each cell population together with gene ontology analysis (biological process). The enrichment score was calculated by determining the expression of a particular gene in a specific cell cluster relative to the mean expression of that same gene across all cell clusters. See also Figure S2.

The identity of individual cells can be determined via scRNA-seq through the analyses of cellular transcriptomes (Grun and van Oudenaarden, 2015). To test the specificity of our selected markers and antibodies, we stained E14.5 brain cells with the pre-screened antibodies, isolated them using FACS and determined cell identity through scRNA-seq with the mCEL-Seq2 protocol (Figure 1E). We defined neural cells using a set of negative makers to ensure we were obtaining the full spectrum of neural cells present in the E14.5 brain (PECAM1^neg^, CD102^neg^, PDGFRβ^neg^, CD45^neg^, CD41^neg^). Endothelial cells, mural cells, and microglia were selected based on positive markers as indicated in Figures 1A, 1B, and 1D. Following scRNA-seq, we filtered for cells with more than 1,500 transcripts, leading to the selection of 625 cells. Through unsupervised clustering of sorted cells using RaceID3 (Grun et al., 2015, Herman et al., 2018), we identified four major cell populations with 12 distinct clusters (Figure 1F). Each of the four major clusters specifically expressed markers of neural cells (*Tubb3*, *Sox11*, *Dcx*), mural cells (*Pdgfrb*, *Rgs5*, *Anpep*), endothelial cells (*Pecam1*, *Kdr*, *Cd93*), or microglia (*Ptprc*, *Itgam*, *C1qc*) (Figure 1G). We next correlated our selected cell surface markers to the identity of cells as determined by unsupervised clustering. We found that our selection criteria allowed us to isolate neural cells, mural cells, endothelial cells, and microglia to between 94% and 100% purity (Figures 2A–2E). Furthermore, each of the populations strongly expressed genes known to be important for their function (Figures 2F–2I, Tables S3–S6), whereas GO-term analyses showed significant enrichment of terms known to functionally correlate with each cell type (Figure 2J). Together, these analyses revealed that we were able to isolate the major cells within the developing brain, namely neural cells, mural cells, endothelial cells, and microglia, to high purity using a FACS-based strategy.

### Identification of Heterogeneity in Neural, Mural, and Endothelial Cell Populations

We next wanted to examine the extent of cellular heterogeneity within each of the cell populations. To this end, we analyzed the clusters within each of the four major cell populations. Neural, mural, and endothelial cell populations contained three distinct clusters each, indicating heterogeneity within each cell type (Figure 1F). On the other hand, only one microglial cluster was detectable, probably because of the small number of cells sequenced (Figure 1F, cluster 5). We identified a number of characteristic genes for each subpopulation using differential gene expression analysis between the clusters (Figure S2). The greatest level of cellular heterogeneity was observed among the three neural clusters, reflecting the number of distinct neural stem and progenitor cells as well as distinct differentiating neurons found within the E14.5 brain (Figures S2A–S2D). Similarly, we detected three distinct cell states in the endothelial and mural cell clusters (Figures S2E–S2J), suggesting that our cell sorting procedure was indeed able to detect and isolate a range of neural, mural, and endothelial cell populations present in the mouse embryonic brain.

### Simultaneous Isolation and Analyses of Neural, Mural, Endothelial, and Microglial Cells Using EMBRACE

**Figure 3:**
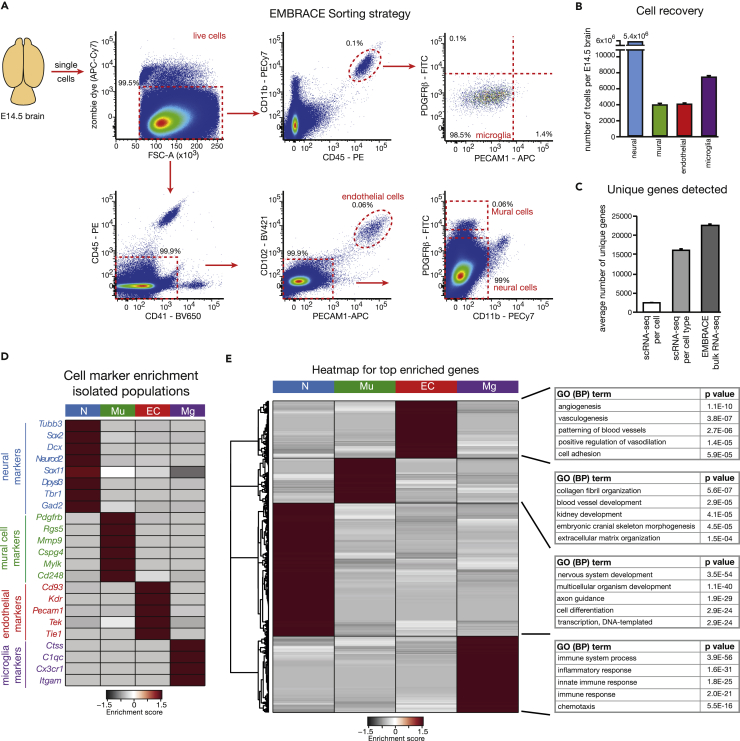
Population-Based Transcriptomic Analyses of Neural, Mural, Endothelial, and Microglial Cells Provide a Rich Cellular Transcriptome (A) EMBRACE sorting strategy with representative gating for the isolation of neural cells, mural cells, endothelial cells, and microglia. (B) Average numbers of cells recovered from each E14.5 brain. Error bars indicate mean ± SEM. N = 6 E14.5 brains for neural cells and N = 11 E14.5 brains for endothelial, mural, and microglial cells. (C) Mean number of unique genes detected per cell and per cell type in the scRNA-seq data, as well as in EMBRACE-based bulk RNA-seq experiments. Error bars indicate mean ± SEM. N = 625 single cells isolated from three independent brains with a minimum of 1,500 unique transcripts; N = 3 wild-type brains for bulk population-based analyses. (D) Enrichment of neural, mural, endothelial, and microglial markers in the four EMBRACE-enriched populations. A comprehensive list of genes enriched in each of the four populations is provided in Tables S7–S10. (E) Gene ontology (biological processes) analyses of the top enriched genes (enrichment >10-fold) in each of the four EMBRACE-isolated cell populations — neural, mural, endothelial, and microglial cells. The enrichment scores in (D) and (E) were calculated by determining the expression of a particular gene in a specific cell type relative to the mean expression of that same gene across all 4 EMBRACE-isolated cell populations. EC - endothelial cells; Mg - microglia; Mu - mural cells; N - neural cells. See also Figure S3.

Although scRNA-seq is an ideal tool for detecting cellular heterogeneity and phenotypic shifts in cell populations, only the most strongly expressed transcripts are typically detected. This may hinder the ability to detect the complete transcriptome of cells and undertake detailed analyses of functionally important lowly expressed genes and transcription factors. For this purpose, we simultaneously collected neural cells (CD45^neg^, CD41^neg^, CD11b^neg^, PECAM1^neg^, CD102^neg^, PDGFRβ^neg^), mural cells (PDGFRβ^high^, PECAM1^neg^, CD102^neg^, CD45^neg^, CD41^neg^, CD11b^neg^), endothelial cells (PECAM1^+^, CD102^+^, CD45^neg^, CD41^neg^, CD11b^neg^, PDGFRβ^neg^), and microglia (CD45^medium^, CD11b^+^, PECAM1^neg^, PDGFRβ^neg^) from E14.5 brain samples by FACS using the markers that we had established (Figure 3A). We refer to this methodology as EMBRACE. Using EMBRACE, we obtained around 5.4 million neural cells, 4,000 mural cells, 4,000 endothelial cells, and 7,000 microglia from each E14.5 brain (Figure 3B). We analyzed the isolated cell population transcriptomes via RNA-seq. Compared with an average of 2,439 unique genes per cell in the single-cell RNA-seq experiments, we detected around 24,000 genes in the bulk population RNA-seq, giving us greater power for downstream analyses (Figure 3C). These observations were consistent with other scRNA-seq studies (Rosenberg et al., 2018, Vanlandewijck et al., 2018, Zeisel et al., 2018), which also detected between 677 and 3,254 unique genes per cell (Figure S3A). The EMBRACE-isolated cell populations were strongly enriched in their respective cell markers (Figure 3D and Tables S7–S10) and showed cell-type-appropriate biological functions in GO-term analyses (Figure 3E), thereby confirming their expected cell identities. Together, these analyses revealed that we could isolate highly enriched neural, mural, endothelial, and microglial cell populations via EMBRACE and detect a much richer transcriptome with bulk RNA-seq compared with scRNA-seq analyses.

### Building a Cell-Cell Interaction Database

**Figure 4:**
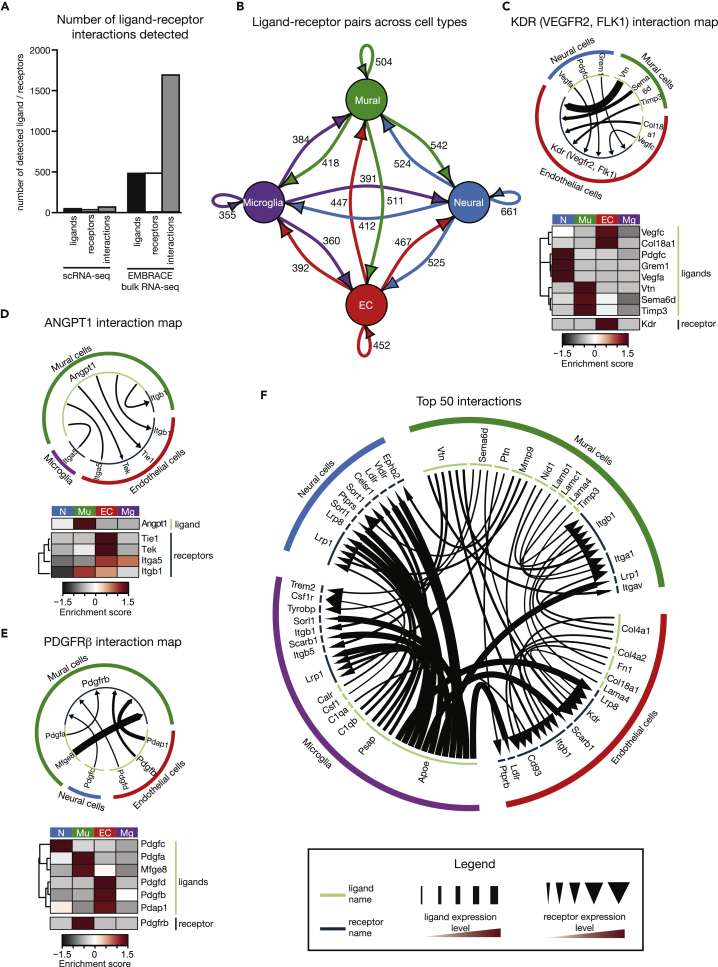
Extensive Inter-Cellular Communication in the Developing Brain (A) Number of detected ligands, receptors, and ligand-receptor pairs in the scRNA-seq dataset compared with transcriptional analyses of bulk isolated cell populations. (B) Map of ligand and receptor interactions between neural, mural, endothelial, and microglial cells based on transcriptomic analyses of EMBRACE-isolated cell populations. Arrow color corresponds to the ligand source, and the numbers indicate the quantity of detected ligand-receptor pairs between the indicated cell types. The comparable interaction map for the scRNA-seq data is provided in Figure S3B. (C) Interaction map of the KDR (VEGFR2, FLK1) receptor network. Expression of *Kdr* is confined to endothelial cells, whereas its ligands are expressed by neural, mural, and endothelial cells. The heatmap represents the enrichment of gene expression in the four different cell types. (D) Interaction map and associated heatmap for the ligand ANGPT1 (Angiopoietin). *Angpt1* is expressed only in mural cells and has corresponding receptors in microglia and endothelial cells. (E) Interaction map and corresponding heatmap of the PDGFRβ receptor network. (F) Top 50 strongest predicted interactions between neural, mural, endothelial, and microglia cells. This list was generated by ranking the absolute expression levels of ligand-receptor pairs across the four cell types and filtering for ligands showing cell-type specific expression. The associated heatmap is provided in Figure S4D. The enrichment score was calculated by determining the expression of a particular gene in a specific cell type relative to the mean expression of that same gene across all four EMBRACE-isolated cell populations. EC – endothelial cells, Mg – microglia, Mu – mural cells, N – neural cells. See also Figures S3 and S4.

To uncover potential communication between neural, mural, endothelial, and microglial cells in the developing mouse brain, we built an *in silico* cell-cell communication network via quantification of ligand-receptor interactions between the four different cell types. We utilized the ligand-receptor pair dataset generated by Ramilowski and co-workers to identify cell-cell interactions (Ramilowski et al., 2015). We filtered the bulk RNA-seq data for genes that showed an average expression of greater than 10 FPKM across all four cell types. Consistent with the greater sensitivity of RNA-seq on bulk-sorted populations, we were able to identify 20-fold more cell-cell interactions in the bulk RNA-seq dataset compared with scRNA-seq data (Figures 4A and S3B–S3D). Indeed, our bioinformatics analyses predicted between 350 and 550 ligand-receptor interactions between each of the four different cell types with a total of 1,710 unique interactions based on the EMBRACE RNA-seq data, including extensive autocrine signaling (Figure 4B and Table S11). We have provided access to the complete inter-cellular communication database via the online “Brain interactome explorer” (https://mpi-ie.shinyapps.io/braininteractomeexplorer/).

We next wanted to test the accuracy of our database by interrogating the presence of well-established cell-cell interactions that are known to occur in the developing brain. VEGF-A from neural cells is known to induce the vascularization of the brain via angiogenesis (Haigh et al., 2003). Consistently, our analyses predicted a substantial interaction between *Vegfa* from neural cells with the VEGF receptor *Kdr* (*Vegfr2*) in endothelial cells (Figure 4C). Similarly, the interaction between the angiopoietin (Angpt) ligands and Tie receptors as well as between platelet-derived growth factors (PDGF) and PDGF receptors is critical for proper vascular development. We could indeed map strong interactions connecting these ligand-receptor pairs (Figures 4D and 4E). In addition, we searched for established communication modules essential for neural cell development. We uncovered widespread WNT and Ephrin signaling centered around neural cells (Figures S4B and S4C), consistent with the critical role of WNTs and Ephrins in neural development (Lisabeth et al., 2013, Mulligan and Cheyette, 2012, Noelanders and Vleminckx, 2017). Together, these examples suggest that our inter-cellular communication network is able to detect important inter-cellular communication modules known to occur in the E14.5 brain.

**Figure 5:**
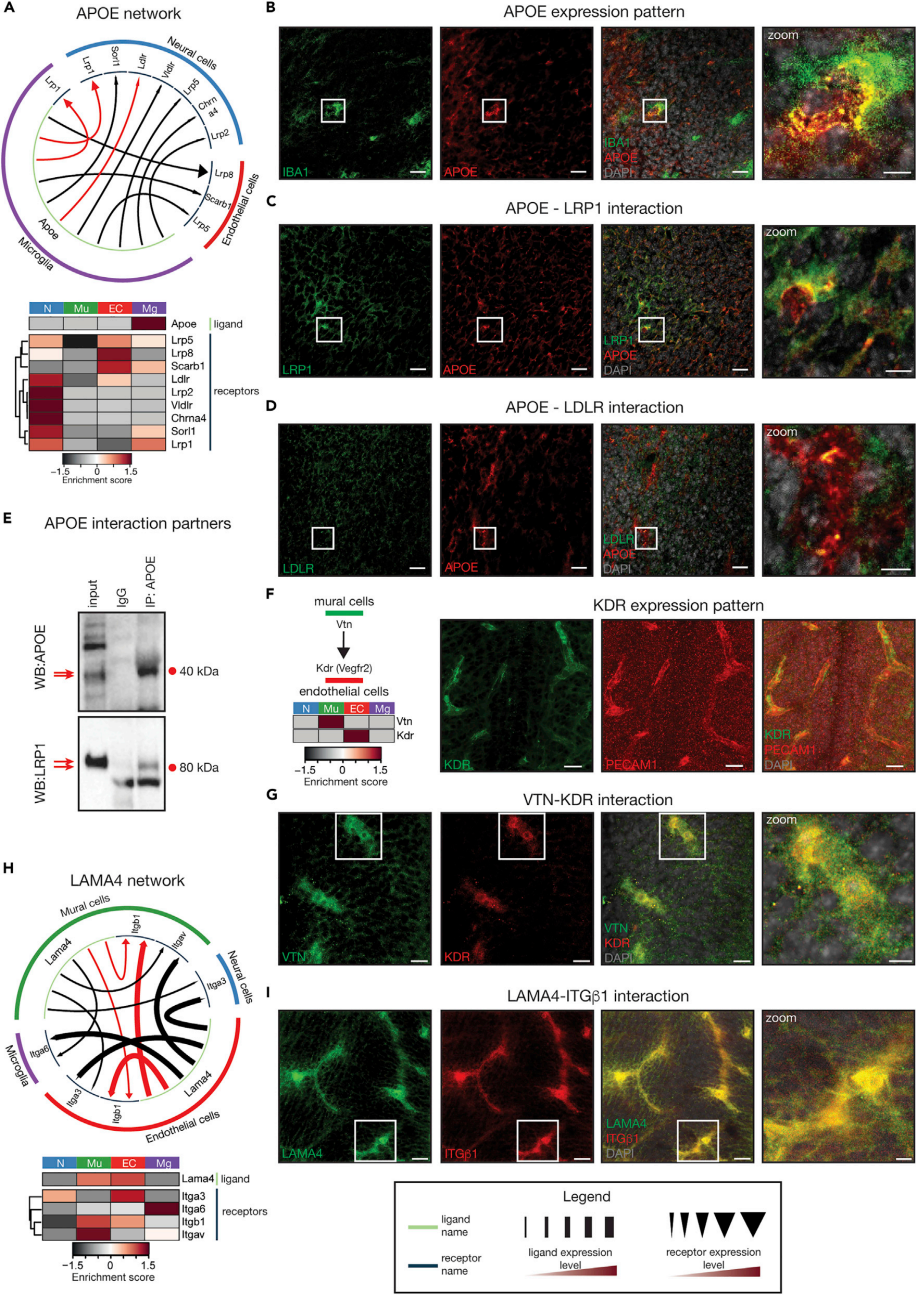
Confirmation of Inter-Cellular Signaling Modules (A) Interaction map of the apolipoprotein E (APOE) network. *Apoe* is strongly expressed in microglia, whereas its receptors are primarily enriched in endothelial and neural cells. The heatmap displays enrichment in gene expression of *Apoe* and its receptors in the different cell populations. Red lines indicate the predicted APOE–LDLR and the APOE–LRP1 interactions that were experimentally tested. (B) Enrichment of APOE protein expression in IBA1-positive microglia in the E14.5 brain. Scale bars are 20 μm in the first three panels and 5 μm in the zoom panel. (C) Immunofluorescence imaging showing overlap of APOE (red) and LRP1 (green) proteins in the E14.5 brain. Scale bars represent 20 μm in the first three panels and 5 μm in the zoom panel. (D) Immunofluorescence showing overlap of APOE (red) and LDLR (green) proteins in sections of the E14.5 brain. Scale bars represent 20 μm in the first three panels and 5 μm in the zoom panel. (E) Co-immunoprecipitation assay showing direct protein interaction between APOE and LRP1. APOE was immunoprecipitated from E14.5 brain lysates and the IP material was subsequently probed with APOE and LRP1 antibodies via western blot analysis. (F) Diagram showing the expected interaction between VTN and KDR. The full KDR interaction network is provided in Figure 4C. The immunofluorescence images show the expected enrichment of KDR protein expression in PECAM1-positive endothelial cells. Scale bars indicate 20 μm. (G) Immunofluorescence imaging of E14.5 brain sections showing overlap between vitronectin (VTN, green) and KDR (red) protein expression. Scale bars indicate 10 μm in the first three panels and 5 μm in the zoom image. (H) Interaction map and corresponding heatmap of the LAMA4 network. The LAMA4 and ITGB1 interaction, highlighted in red, was tested by immunofluorescence analyses. (I) Immunofluorescence images showing overlap between LAMA4 (green) and ITGβ1 (red) proteins in the E14.5 brain. Scale bars indicate 20 μm in the first three panels and 5 μm in the zoom image. The enrichment score was calculated by determining the expression of a particular gene in a specific cell type relative to the mean expression of that same gene across all four EMBRACE-isolated cell populations. EC - endothelial cells; IP - immunoprecipitation; Mg - microglia; Mu - mural cells; N - neural cells; WB - western blot. See also Figure S4.

Having established that we could detect well-established cell-cell interactions in our dataset, we next focused on detecting potentially novel and previously overlooked communication modules within the developing brain. We mapped the interaction of the top 50 expressed ligand-receptor pairs, for which the ligand expression was enriched in at least one cell population (Figures 4F and S4D). Cells with the highest broadcasting capacity were microglia followed by mural cells. The strongest interactions were detected between microglia and neural cells and were underpinned by APOE signaling (Figures 4F and S4D). Although the importance of APOE signaling and *APOE* allele variants in Alzheimer disease is well established (Farrer et al., 1997, Poirier et al., 1993, Strittmatter et al., 1993), the importance of *Apoe* in development is comparatively poorly understood. Our transcriptomic analysis showed high levels of *Apoe* expression in microglia at E14.5 (Figure 5A). We confirmed the strong enrichment of APOE expression in IBA1^+^ microglia via immunofluorescence (Figure 5B). Consistent with the fact that APOE is a secreted protein (Bu, 2009), we also observed widespread APOE signal in the E14.5 brain (Figure 5B). We next tested whether APOE could interact with its known receptors LRP1 and LDLR in the E14.5 brain. Through immunofluorescence, we found the APOE expression pattern to overlap with LRP1 and LDLR signals (Figures 5C, 5D, and S4E). In addition, immunoprecipitation of endogenous APOE was able to pull down LRP1 in lysates of the E14.5 brain (Figure 5E), highlighting the existence of significant APOE signaling activity in the developing brain. These data suggest that microglia-expressed APOE is likely to have an important role in brain development through its extensive signaling to embryonic neural and endothelial cells.

Mural cells showed strong expression of secreted proteins that are not well studied in the context of neural development. The most prominent was vitronectin (*Vtn*), which is thought to signal via integrins as well as KDR (Murphy and Stupack, 2010, Wang et al., 2015). Consistent with previous work (Seiffert et al., 1995), we observed that VTN expression is confined to mural cells in the E14.5 brain (Figure 5F, left panel and S4D). The significance of these observations is currently unknown. Nevertheless, work in human umbilical cord vascular endothelial cells (HUVEC) has suggested that glycated VTN is able to inhibit the KDR receptor as well as the outgrowth and migration of endothelial cells (Wang et al., 2015). Consistently, our analyses suggest strong *Vtn* signaling from mural cells to *Kdr* in endothelial cells (Figure 5F). We were able to confirm the endothelial-specific expression of KDR as well as the interaction between VTN and KDR in the E14.5 brain using immunofluorescence analyses (Figures 5F and 5G). Although the importance of this communication module in the developing brain is yet to be established, this interaction is consistent with the role of mural cells in controlling aspects of endothelial cell development.

Mural cells also strongly expressed pleiotrophin (*Ptn*), a peptide showing high expression during development and in diseased states (Li et al., 1990, Poulsen et al., 2000, Takeda et al., 1995, Yeh et al., 1998). PTN can signal through a multitude of receptors, including integrins, N-syndecan, and receptor protein tyrosine phosphatases (PTPR) (Gonzalez-Castillo et al., 2014). In neural injury models, PTN is strongly upregulated and acts in a neuro-protective manner while promoting outgrowth of neurites (Mi et al., 2007). The precise contribution of PTN during the developmental time frame remains unknown. However, it is likely to play an important role given that *Ptn* knockout animals display neurological symptoms including increased anxiety, reduced social interaction, and a reduction in layer IV of the cortex (Krellman et al., 2014).

The most prominent ligands expressed by endothelial cells were non-canonical ligands of extracellular matrix proteins, including collagens and laminin subunit A4 (*Lama4*), which act primarily on endothelial cells as well as mural cells (Figure 4F). LAMA4 is found in the extracellular matrix surrounding endothelial cells and binds to integrin β1 (ITGB1) (Gonzalez et al., 2002). In the context of the E14.5 brain, *Itgb1* displayed the highest expression in mural cells, whereas LAMA4 was most strongly expressed in endothelial cells (Figure 5H). We found a strong overlap between LAMA4 and ITGβ1 protein expression in immunofluorescence stainings (Figure 5I). Although *Lama4* deletion is known to cause defects in angiogenesis (Stenzel et al., 2011), the importance of the cross talk between endothelial LAMA4 and mural cell ITGB1 during neural vasculature development remains unclear.

Together, these examples highlight the versatility of our approach in facilitating the identification of potentially new and under-appreciated inter-cellular communication modules within the developing brain.

## Discussion

In the current study, we set out to establish a method for the simultaneous isolation of neural, mural, endothelial, and microglial cells and subsequently apply this method to map the cell-cell communication networks that exist in the developing mouse brain. We show here that we could enrich each of the four cell populations to more than 94% purity using EMBRACE. Undertaking a direct comparison of single cell and bulk FACS-purified transcriptomes, we show that around 20-fold more ligand-receptor pairs are detectable in the bulk FACS-purified populations. Using an *in silico* approach, we map the cell-cell communication networks and chart the E14.5 brain interactome, revealing extensive communication between neural, mural, endothelial, and microglial cells. We experimentally confirmed selected interactions from the top 50 list, including APOE-LRP1, APOE-LDLR, VTN-KDR, and LAMA4-ITGβ1, emphasizing the biological relevance of our database.

Since cells *in vivo* are present in a complex environment, it is important to study gene function in specific cell types in the context of the whole organ or even the organism. Using single cell or nucleus RNA-seq analysis, inter-cellular communication databases have been generated for the heart (Skelly et al., 2018), kidney (Wu et al., 2019), liver organoids (Camp et al., 2017), as well as tumors isolated from mouse models (Kumar et al., 2018) and human patients (Puram et al., 2017, Zhou et al., 2017). Although single cell RNA-seq is powerful in the identification of rare cell types and detection of heterogeneity between similar cell populations, bulk RNA-seq analyses on cell populations provides a much richer transcriptome. Thus, using a combination of both scRNA-seq and bulk population-based RNA-seq provides a much more comprehensive picture of cell function compared with either technique alone. The disadvantage of a population-based RNA-seq approach is the time-consuming task of identifying and verifying cell-type specific markers that are critical for the isolation of near-pure cell populations. In contrast, cell identity can be retrospectively assigned in scRNA-seq datasets based on the cellular transcriptome. However, not all genomic techniques are currently amenable to single cell technologies and require the isolation of larger cell populations. To this end, we have established EMBRACE, a method to simultaneously isolate neural, mural, endothelial, and microglial cells to high purity. This sorting methodology will be particularly useful to researchers conducting experiments that are not yet compatible with single cell analyses, such as 3D chromatin conformation capture or chromatin immunoprecipitation (ChIP). Furthermore, given the simplicity of EMBRACE, it can easily be applied to study cell-cell communication in cell-type specific knockout mouse models.

In contrast to EMBRACE, more complex techniques have been applied to isolate different cell types from the adult mouse brain. Through immunopanning, transgenic animal models, as well as FACS, Zhang and co-workers have enriched and transcriptionally analyzed endothelial cells, microglia, progenitor and mature oligodendrocytes, neurons, and astrocytes (Zhang et al., 2014, Zhang et al., 2016) and reported significant differences in transcriptional splicing between these cell types (Zhang et al., 2014). Although it is difficult to ascertain whether all cellular subtypes survive the purification procedure, these datasets nevertheless provide an excellent resource for exploring the transcriptional complexity of cell types in the adult brain. In contrast, we have focused here on the embryonic brain to determine inter-cellular communication modules that are likely important for neural development. We identified a total of 1,710 interactions, many of which are likely to play an important role during development (Figure 4B and Table S11, https://mpi-ie.shinyapps.io/braininteractomeexplorer/).

Among the strongest interactions, we found microglia-expressed *Apoe* to mediate communication with neural, endothelial, and mural cells in the E14.5 brain (Figures 4F and 5A–5E). APOE is highly abundant in the brain and is involved in lipid transport, clearance of lipoproteins, and cholesterol homeostasis (Hauser et al., 2011, Huang and Mahley, 2014). *Apoe* knockout mice kept on a high-fat diet show significant accumulation of lipids in the brain (Walker et al., 1997). At least in mouse models, APOE cannot cross the BBB (Liu et al., 2012) and must therefore be produced locally. Our analyses show that microglia are the major producers of APOE in the E14.5 brain (Figures 5A and 5B). There are some indications that APOE may be important in brain development. The *APOE* ϵ4 allele variant is the best-known genetic risk factor associated with Alzheimer disease (Farrer et al., 1997, Poirier et al., 1993, Strittmatter et al., 1993), and infants carrying this variant show delayed myelination and gray matter development in brain regions typically affected by Alzheimer disease (Dean et al., 2014). In addition to extensive signaling between microglia-derived APOE and neural cells, our analyses revealed interactions between APOE and its reported receptors in vascular cells (Figure 4F). These interactions are also likely to be important during development, as mice lacking *Apoe* display increased permeability of neural vasculature but not of vessels from other organs (Hafezi-Moghadam et al., 2007, Methia et al., 2001). It is currently unknown which molecular mechanisms drive this specific defect in neural vasculature and how *Apoe* deficiency impacts neural vascular function. Together with our cell-cell interactome, these studies highlight the potential importance of APOE during the developmental time frame and encourage the investigation of APOE during early brain development. In addition to APOE, our dataset provides a resource of 1,710 total inter-cellular interactions that are likely to play important roles during the neural developmental time frame.

In summary, we describe here EMBRACE, a method to simultaneously isolate neural, mural, endothelial, and microglial cells to high purity. This simple method will allow researchers to explore inter-cellular interactions in their mouse models of choice. Using EMBRACE, we isolated, transcriptionally analyzed, and built an inter-cellular communication network in the E14.5 brain that revealed 1,710 unique ligand-receptor relationships (https://mpi-ie.shinyapps.io/braininteractomeexplorer/). Our dataset and brain communication network underline the richness of inter-cellular communication present in the developing brain and provide a comprehensive resource that will allow the field to dissect the importance of selected receptors and ligands in the context of neural development.

### Limitations of the Study

In the current study, we establish the EMBRACE methodology that we use to analyze inter-cellular communication in the developing mouse brain. Given the complexity of adult brains, which consist of extensive neural processes that are prone to damage during dissociation, the EMBRACE procedure may not be successful in isolating neural cells from the adult brain. However, EMBRACE is still likely to be efficient in isolating endothelial, mural, and microglial cells from the adult brain, as they express the same cell surface markers as their embryonic counterparts and are resistant to harsh dissociation regimens.

Using the EMBRACE technique, we isolated neural, mural, endothelial, and microglial cells from the E14.5 brain and built an inter-cellular interactome based on the transcriptional profiles of each cell type. Our database is built on gene expression levels in neural, mural, endothelial, and mural cells and uses a collated list of ligand-receptor interactions (Ramilowski et al., 2015), which is based on diverse cell types. Thus, it will be prudent for researchers to experimentally confirm their interactions of interest in a similar fashion as we have done for APOE-LDLR, APOE-LRP1, VTN-KDR, and LAMA4-ITGβ1 in the E14.5 brain.

## Methods

All methods can be found in the accompanying Transparent Methods supplemental file.

## Data and Code Availability

The Brain Interactome Explorer is available at https://mpi-ie.shinyapps.io/braininteractomeexplorer/.

Raw sequencing data have been uploaded to GEO and are available under GSE133079.

## Supplemental Information

Document S1. Transparent Methods, Figures S1–S4, and Tables S1–S10

Table S11. Complete List of Mapped Cell-Type Specific Interactions amongst the EMBRACE-Enriched Populations, Related to Figure 3 A value of 0 designates the absence of an interaction, whereas 1 designates the presence of an interaction. A searchable database containing the strength of predicted interactions can be viewed at https://mpi-ie.shinyapps.io/braininteractomeexplorer/.
