# Supplementary material for: Systematic Identification of Cell-Cell Communication Networks in the Developing Brain
Source: iScience. 2019 Oct 17;21:273–87. doi: 10.1016/j.isci.2019.10.026 (PMC6838536; doi:10.1016/j.isci.2019.10.026)
Supplement: Document S1. Transparent Methods, Figures S1–S4, and Tables S1–S10 [file mmc1.pdf]

ISCI, Volume *21*

Supplemental Information

Systematic Identification of Cell-Cell Communication Networks in the Developing Brain

Bilal N. Sheikh, Olga Bondareva, Sukanya Guhathakurta, Tsz Hong Tsang, Katarzyna Sikora, Nadim Aizarani, Sagar, Herbert Holz, Dominic Grün, Lutz Hein, and Asifa Akhtar

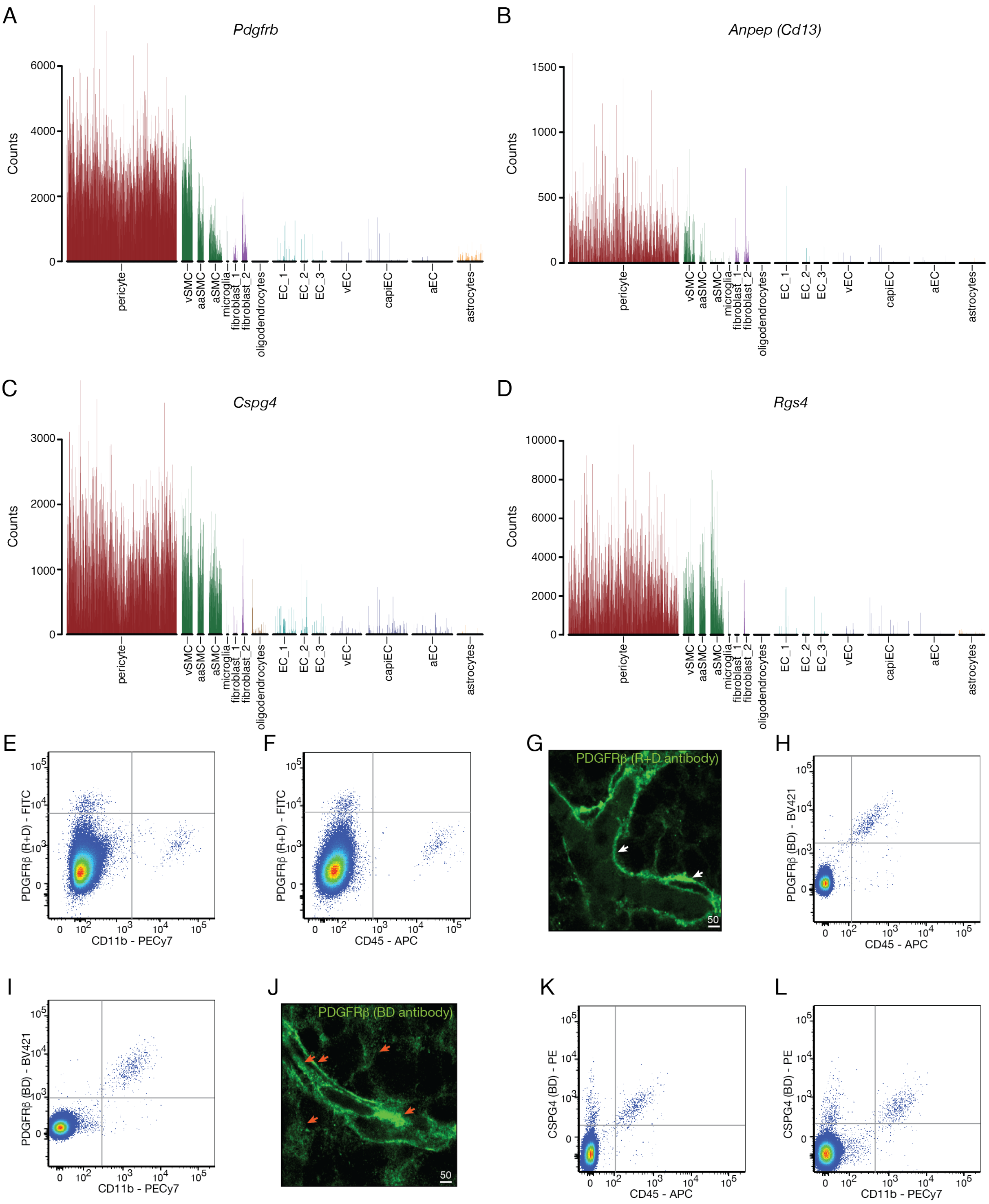

Figure S1

Screening for potential mural cell markers and antibodies.

Related to Figure 1

(A-D) Expression of potential mural cell markers in the published scRNA-seq database by the Betscholtz laboratory (Vanlandewijck et al., 2018). **(A)** *Pdgfrb* showed consistently high expression in all pericytes and smooth muscle cells. PDGFRβ is expressed on the cell surface making it ideal for downstream use. **(B)** *Anpep* (*Cd13*) showed weak expression that was undetectable in some pericytes. While this may be due to the low sensitivity of scRNA-seq, it also suggests that CD13 expression may be heterogeneous within the mural cell population resulting in some pericytes and

smooth muscle cells remaining undetected. **(C)** *Cspg4* displayed strong expression in pericytes and smooth muscle cells and thus was shortlisted for downstream testing. **(D)** While *Rgs4* showed strong expression in pericytes and smooth muscle cells, it is an intracellular marker and thus not conducive to FACS. **(E)** Flow cytometry plot comparing the expression of PDGFRβ (R+D antibody) and the microglia-specific marker CD11b. PDGFRβ (R+D) antibody did not show any cross reactivity with CD11b. **(F)** Flow cytometry plot showing no overlap in staining with the PDGFRβ (R+D) antibody and the microglia and hematopoietic marker CD45. **(G)** Immunofluorescence staining using the PDGFRβ (R+D) antibody showing clear peri-vascular staining (white arrows) in the E14.5 brain cortex. Also see Figure 1C. **(H-I)** Flow cytometry plot showing cross-reactivity between the Becton Dickinson (BD) PDGFRβ antibody and microglial markers **(H)** CD45 and **(I)** CD11b. **(J)** Immunofluorescence staining using the PDGFRβ (BD) antibody showing unexpected intracellular staining in vascular cells as well as widespread background signal outside of the vasculature (marked by red arrows). **(K-L)** Flow cytometry plot showing cross-reactivity between the Becton Dickinson (BD) CSPG4 antibody and microglial markers **(K)** CD45 and **(L)** CD11b.
Based on these analyses, the BD PDGFRβ and CSPG4 antibodies were not used and all experiments utilised the PDGFRβ antibody from R+D.
N = 3 to 10 replicates per experiment in E-L.

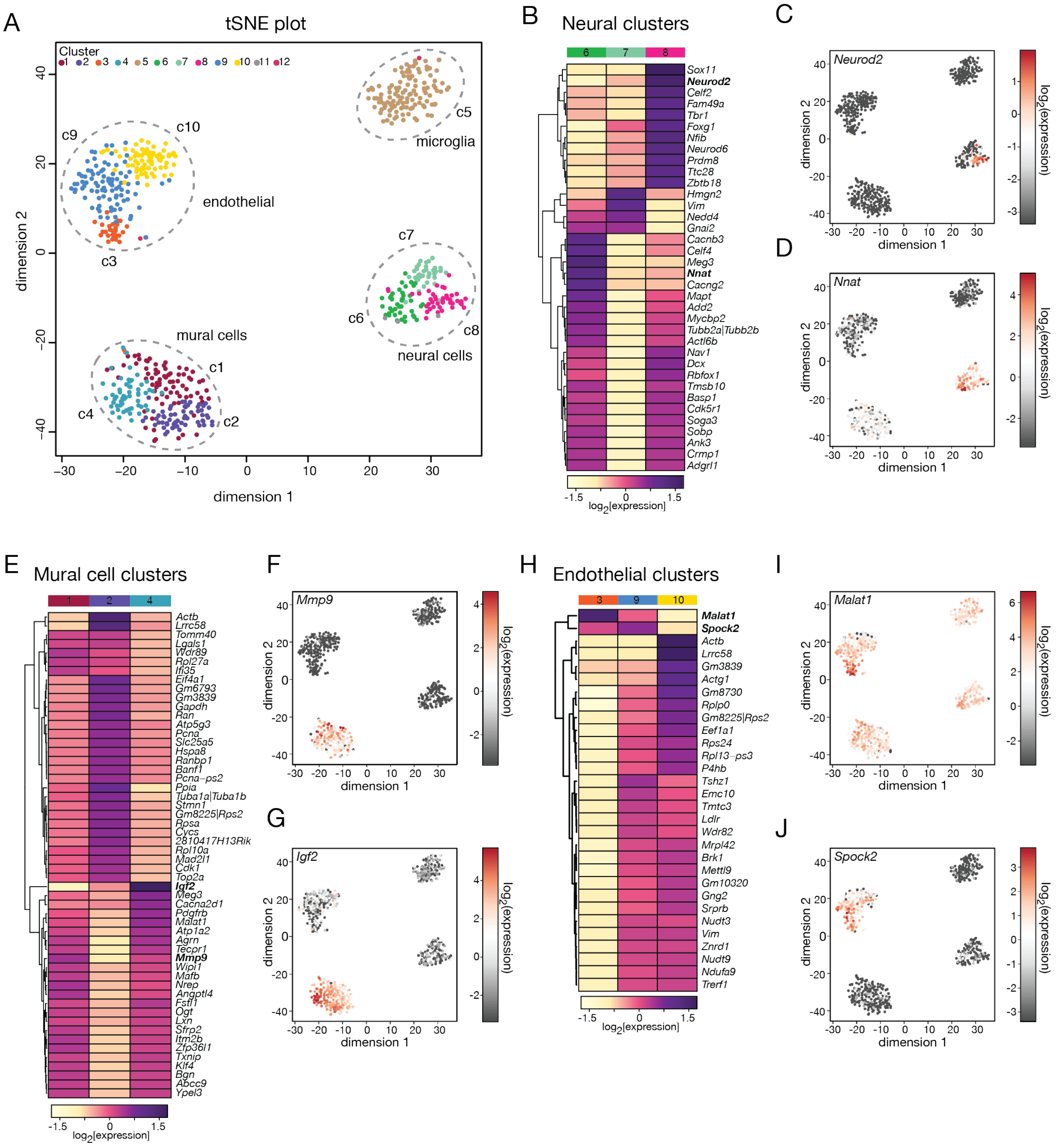

Figure S2

Comparison between subpopulations of neural cells, mural cells and endothelial cells.

Related to Figures 1 and 2

(A) An unbiased t-distributed stochastic neighbour embedding (tSNE) plot of all sorted cells, regardless of the cell surface marker used for their isolation, depicting 12 total clusters encompassing the four major cell populations: neural cells (clusters 6 to 8), mural cells (clusters 1, 2 and 4) endothelial cells (clusters 3, 9 and 10) and microglia (cluster 5). While each of the neural, mural and endothelial populations could be split into 3 three sub-clusters, only one cluster for microglia was evident. **(B)** Heatmap depicting differential gene expression between the three main sub-populations of the neural population. The $\log_2$(normalised counts) value is provided for the top differentially expressed genes that display an enrichment score of more than 1. **(C)** tSNE plot showing *Neurod2* mRNA enrichment in differentiating neurons (cluster 8) compared to the stem / progenitor stem cell population (cluster 6). **(D)** tSNE plot showing enrichment of *Nnat* in cluster 6. **(E)** Heatmap depicting differential gene expression between the three sub-populations of mural cells isolated based on

PDGFRβ expression. The log_2(normalised counts) value is provided for the top differentially expressed genes that display an absolute enrichment score of more than 0.6. **(F)** tSNE plot displaying expression of *Mmp9*, which is enriched in clusters 1 and 4, while lowly expressed in cluster 2. **(G)** tSNE plot of *Igf2* expression. *Igf2* shows strongest expression in cluster 4 and is significantly lower in clusters 1 and 2. **(H)** Heatmap depicting differential gene expression between the sub-populations of endothelial cells isolated based on PECAM1 and CD102 expression. The log_2(normalised counts) value is provided for the top differentially expressed genes that display an absolute enrichment score of more than 1. **(I)** tSNE plot showing *Malat1* expression. While *Malat1* is widely expressed, it shows the highest gene expression in cluster 3 of endothelial cells. **(J)** tSNE plot showing gradient of *Spock2* gene expression.

The enrichment score was calculated by determining the expression of a particular gene in the given cell cluster relative to the mean expression of that same gene across all clusters.

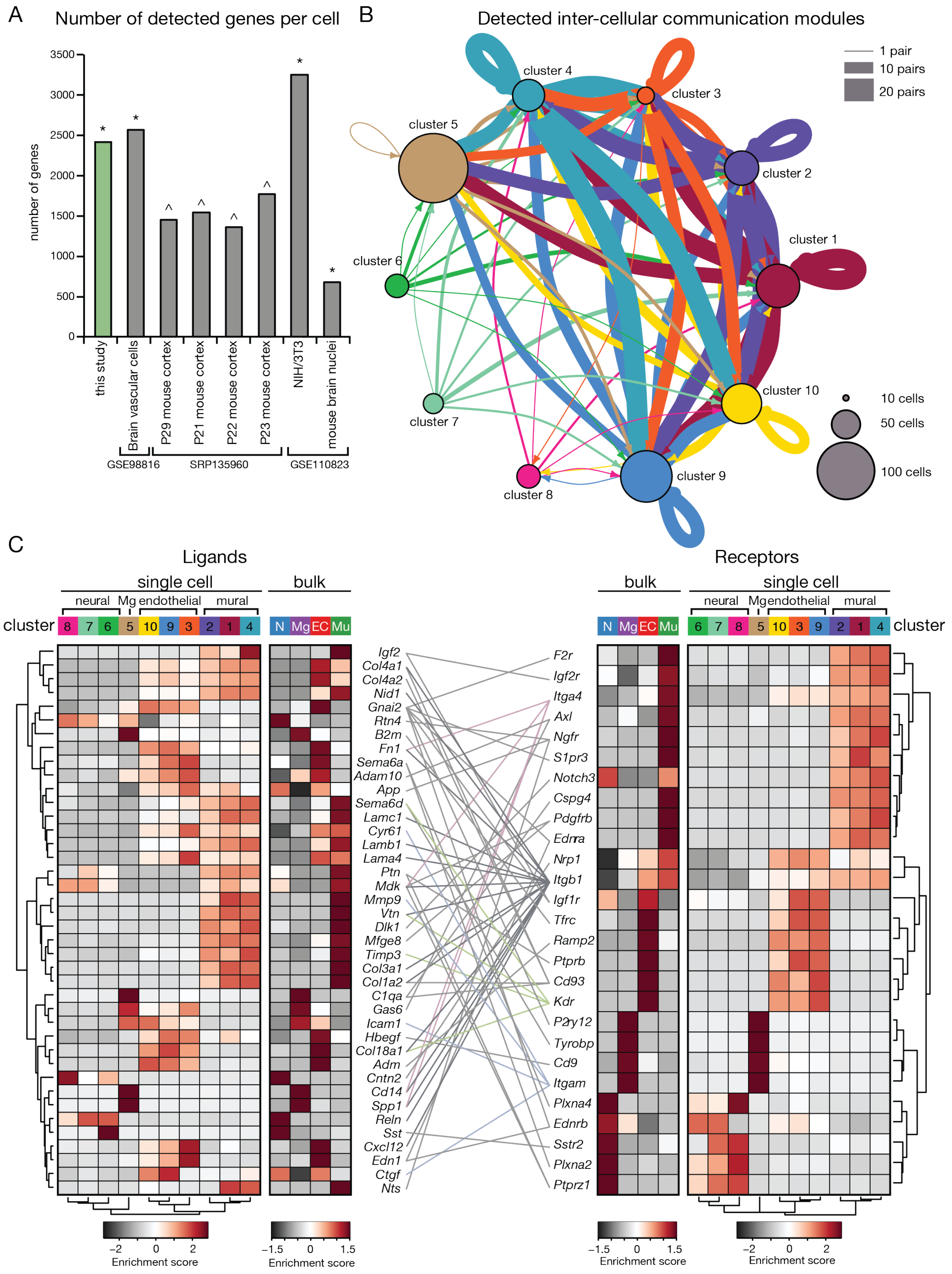

Figure S3

Cell-cell communication networks between neural, mural, endothelial and microglial cell clusters detected in the scRNA-seq dataset.

Related to Figures 3 and 4

(A) Comparison of the number of unique genes detected in scRNA-seq datasets. The GSE98816 dataset is associated with (Vanlandewijck et al., 2018), the SRP135960 dataset with (Zeisel et al.,

2018) and the GSE110823 dataset with (Rosenberg et al., 2018). * indicate the mean number of genes detected per cell, while ^ represents the median number of genes detected per cell. Note that at greater, saturated sequencing depth (>500,000 reads per cell), Rosenberg et al. (2018) report a mean of 4497 detected genes per NIH/3T3 cell and 2055 genes per brain nucleus. **(B)** Inter-cellular interaction network of ligands and receptors between the 10 major clusters identified from the scRNA-seq data. A total of 80 ligands and 61 receptors were expressed in at least one cell cluster without taking into consideration the existence of the complete pair. 61 pairs encompassing 40 ligands and 27 receptors were detected in the whole database. The ligand – receptor interactions were based on the dataset curated by (Ramilowski et al., 2015). The thickness of lines connecting the respective clusters indicates the number of ligand – receptor interactions between the clusters. The size of the cluster circle represents the number of cells identified in the particular cluster. **(C)** Heatmaps showing the enrichment of ligands and receptors in each cluster of the scRNA-seq data as well as in bulk EMBRACE-isolated populations. Lines connecting ligand and receptor pairs depict the interaction between them.

The enrichment score was calculated for scRNA-seq data by determining the expression of a particular gene in the given cell cluster relative to the mean expression of that same gene across all clusters. For bulk RNA-seq analyses, the enrichment score was quantified by determining the expression of a particular gene in a specific cell type relative to the mean expression of that same gene across all 4 EMBRACE-isolated cell populations.

EC – endothelial cells, Mg – microglia, Mu – mural cells, N – neural cells

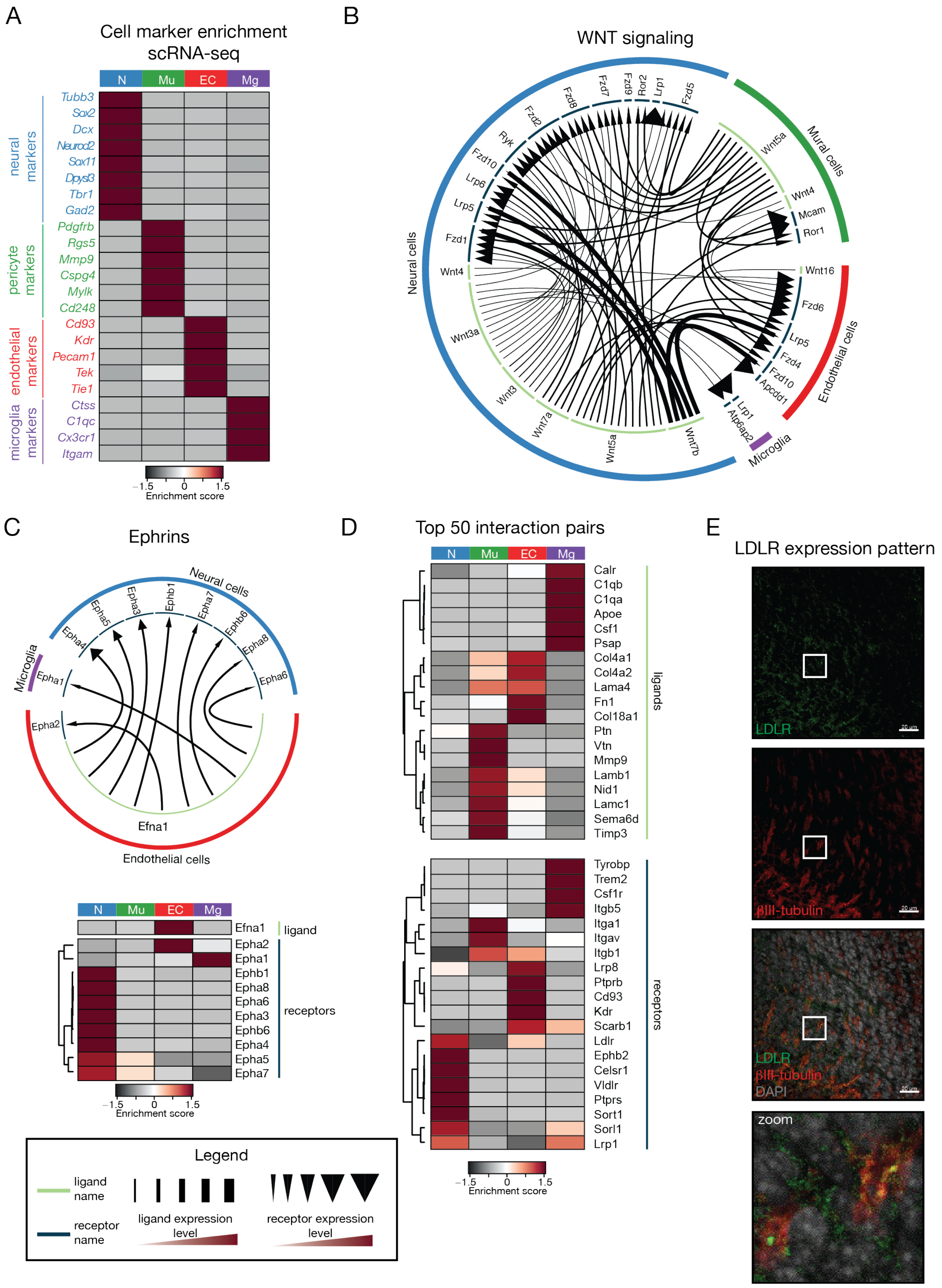

Figure S4

Key communication networks in neural, mural, endothelial and microglial cell populations

Related to Figures 4 and 5

(A) Enrichment of cell type markers in the scRNA-seq dataset for each cell type. **(B)** Interaction ligand - receptor map for WNT signaling based on transcriptomic analyses on bulk sorted populations. Note the high number of interactions involving neural cells. **(C)** Interaction map for Ephrin A1 (*Efna1*), which is highly enriched in endothelial cells, and its receptors, which are primarily expressed in neural cells. The heatmap displays enrichment in gene expression of Ephrin A1 and its receptors in the different

cell populations based on the enrichment score. **(D)** Heatmap based on the interaction map provided in Figure 4F. The heatmap displays gene expression of the 50 highest scoring ligand-receptor pairs bioinformatically mapped in the developing brain. The enrichment of each ligand and receptor is shown based on the enrichment score in neural, mural, endothelial and microglial cells. **(E)** Immunofluorescence images showing widespread expression of LDLR, especially in βIII-tubulin positive neurons in the E14.5 brain. Scale bars indicate 20 μm.
The enrichment score was calculated by determining the expression of a particular gene in a specific cell type relative to the mean expression of that same gene across all 4 EMBRACE-isolated cell populations.
EC – endothelial cells, Mg – microglia, Mu – mural cells, N – neural cells

Table S1

Related to Figure 1

Comparison of methods used for the dissociation of E14.5 brains. The scale spans “-” not detected, to “++++” present at highest levels. “Clumpiness” and “Digestion” were determined by the fraction of cells failing to pass through a 100 µm filter after digestion. Ease of digestion was determined by the amount of manual pipetting required to dissociate the tissue until no obvious tissue clumps were visible.

	No enzyme	Collagenase Dispase	Pancreatin Trypsin	Liberase only	Liberase + Dnase I
**Ease of dissociation**	++++	++	++++	+++	+++
**Clumpiness**	-	++	+++	++	-
**Digestion**	++++	++	+++	+++	++++
**Survival (%)***	17 ± 1.7	39.5 ± 2.2	55.9 ± 1.4	46.6 ± 2.6	67.8 ± 0.7
**Median Fluoresence Intensity (MFI, PECAM1)**	464	308	94	421	not assessed

* proportion of live FACS events as a proportion of total sorted events

Table S2

Related to Figures 1 to 5

List of antibodies used and their specificity. Antibodies shown in red were found to be specific and used in this study. Concentrations provided are for use in immunofluorescence. For Western Blot analysis, antibodies were used at 1:1000. For FACS, the secondary Alexa488-conjugated anti-goat IgG (A11055 ThermoFisher, 1:400) was used to detect the primary PDGFRβ (R+D systems) antibody.

Antibody	Code and Provider	Concentration	Specific?
APOE	Polyclonal 178479 Merck	1:250	Yes
β-III tubulin	clone 5G8 Promega	1:1000	Yes
CD11b	clone M1/70 BD	1:300	Yes
CD13	clone EM15 eBioscience	1:100	Yes, but low signal
CD41	clone MWReg30 BD	1:200	Yes
CD45	clone 30-F11 BD	1:300	Yes
IBA1	EPR16588 (ab178846) Abcam	1:1000	Yes
ICAM2 (CD102)	clone 3C4(mIC2/4) BD	1:250	Yes
KDR (FLK1, VEGFR2)	clone D-8 (sc-393163) Santa Cruz	1:200	Yes
ITGB1	Polyclonal sc-8978 Santa Cruz	1:200	Yes
LAMA4	CL3183 (ab242198) Abcam	1:200	Yes
LDLR	EPY1553Y (ab52818) Abcam	1:250	Yes
LRP1	EPR3724 (ab92544) Abcam	1:250	Yes
NG2 (CSPG4)	clone 9.2.27 BD	1:100	No
NG2 (CSPG4)	Polyclonal AB5320 Millipore	1:100	No
PDGFRβ (CD140b)	clone 28D4 BD	1:200	No
PDGFRβ (CD140b)	Polyclonal AF1042 R+D systems	1:200	Yes
PECAM1 (CD31)	clone 390 eBioscience	1:250	Yes
PECAM1 (CD31)	Polyclonal ab28364 Abcam	1:100	Yes
VTN	EP873Y (ab45139) Abcam	1:200	Yes

Table S3

Related to Figure 2

List of genes most strongly enriched in neural clusters of the scRNA-seq dataset.

Gene	base Mean	Base MeanA	Base MeanB	fold-enrichment	log2 [enrichment]	*p* value	FDR
Dpysl3	1.19	0.13	2.24	17.68	4.14	*6.70E-130*	1.90E-126
Rtn1	1.06	0.13	2.00	15.64	3.97	*6.18E-112*	1.50E-108
Tubb3	0.91	0.11	1.71	15.46	3.95	*4.31E-95*	8.63E-92
Sox11	4.10	0.54	7.65	14.16	3.82	*0.00E+00*	0.00E+00
Elavl3	0.78	0.11	1.45	12.94	3.69	*7.76E-76*	1.15E-72
Stmn2	0.83	0.12	1.54	12.83	3.68	*9.97E-80*	1.79E-76
Dcx	0.67	0.10	1.23	11.68	3.55	*6.39E-62*	7.76E-59
Cd24a	1.00	0.16	1.83	11.25	3.49	*1.52E-90*	2.88E-87
Nsg2	0.64	0.11	1.17	10.86	3.44	*2.16E-57*	2.37E-54
Bcl11b	0.59	0.11	1.07	9.55	3.26	*2.85E-49*	2.55E-46
Nnat	3.53	0.68	6.38	9.38	3.23	*0.00E+00*	0.00E+00
Foxg1	0.55	0.11	0.99	9.27	3.21	*1.66E-45*	1.31E-42
Elavl4	0.58	0.12	1.05	9.14	3.19	*9.49E-48*	7.69E-45
Mapt	0.60	0.12	1.09	9.05	3.18	*9.61E-49*	8.39E-46
Map1b	2.02	0.41	3.62	8.86	3.15	*5.05E-154*	2.46E-150
Gpm6a	0.54	0.12	0.97	8.39	3.07	*8.14E-43*	5.90E-40
Map2	0.61	0.13	1.10	8.37	3.07	*7.11E-48*	5.90E-45
Gap43	0.50	0.11	0.90	8.29	3.05	*4.32E-39*	2.59E-36
Sbk1	0.51	0.11	0.90	8.22	3.04	*1.25E-39*	7.86E-37
Neurod6	0.48	0.11	0.85	7.95	2.99	*1.03E-35*	5.33E-33
Igfbpl1	0.46	0.10	0.81	7.85	2.97	*7.63E-35*	3.71E-32
Lhx2	0.47	0.11	0.83	7.67	2.94	*3.51E-35*	1.79E-32
Meis2	0.46	0.11	0.81	7.46	2.90	*1.01E-32*	4.28E-30
Pou3f3	0.46	0.11	0.80	7.45	2.90	*1.01E-32*	4.28E-30
Sox2	0.49	0.12	0.86	7.38	2.88	*5.39E-35*	2.66E-32
Kif5c	0.44	0.11	0.78	7.30	2.87	*1.67E-31*	6.67E-29
Ina	0.45	0.11	0.79	7.23	2.85	*1.01E-32*	4.28E-30
Nfib	1.75	0.43	3.06	7.05	2.82	*6.69E-117*	1.75E-113
Elavl2	0.43	0.11	0.76	6.90	2.79	*3.89E-30*	1.52E-27
Miat	0.42	0.11	0.74	6.87	2.78	*1.93E-29*	7.11E-27
Dclk1	0.42	0.11	0.73	6.85	2.78	*2.03E-28*	7.26E-26
Trim2	0.43	0.11	0.76	6.84	2.77	*8.27E-30*	3.09E-27
Tubb2a|Tubb2b	2.52	0.65	4.38	6.73	2.75	*3.10E-147*	1.32E-143
37865	0.40	0.10	0.70	6.73	2.75	*3.22E-27*	1.10E-24
Pak3	0.43	0.11	0.74	6.52	2.70	*3.66E-28*	1.30E-25
Bcl11a	0.54	0.14	0.93	6.46	2.69	*1.63E-34*	7.81E-32
Runx1t1	0.42	0.11	0.73	6.34	2.67	*3.64E-27*	1.22E-24
Neurod2	0.38	0.10	0.65	6.29	2.65	*5.11E-25*	1.58E-22
Celf2	1.19	0.33	2.05	6.22	2.64	*4.27E-73*	6.05E-70
Celf4	0.39	0.11	0.68	6.21	2.64	*4.23E-25*	1.32E-22
Ank3	0.41	0.11	0.70	6.19	2.63	*5.56E-26*	1.77E-23
Nfix	0.48	0.13	0.82	6.12	2.61	*4.36E-30*	1.67E-27
Nsg1	0.42	0.12	0.72	6.08	2.60	*4.48E-26*	1.44E-23
Gria2	0.37	0.10	0.63	6.00	2.59	*9.53E-23*	2.64E-20
Cntn2	0.40	0.12	0.69	5.89	2.56	*6.65E-25*	2.04E-22
Cdk5r1	0.53	0.15	0.91	5.89	2.56	*4.79E-32*	1.94E-29
Dlx1	0.36	0.11	0.61	5.81	2.54	*8.88E-22*	2.34E-19
Pbx1	0.88	0.26	1.49	5.74	2.52	*1.06E-50*	1.06E-47
Crmp1	0.57	0.17	0.97	5.67	2.50	*7.08E-33*	3.13E-30
Stmn1	2.63	0.80	4.47	5.61	2.49	*4.57E-130*	1.41E-126
Basp1	2.11	0.64	3.58	5.58	2.48	*1.24E-111*	2.81E-108
Tagln3	0.47	0.15	0.79	5.38	2.43	*3.24E-26*	1.05E-23
Soga3	0.37	0.12	0.63	5.32	2.41	*7.89E-21*	1.99E-18
Kif21a	0.37	0.12	0.62	5.23	2.39	*2.29E-20*	5.49E-18
Fez1	0.34	0.11	0.57	5.21	2.38	*4.40E-19*	9.72E-17
St8sia2	0.35	0.11	0.59	5.20	2.38	*2.74E-19*	6.22E-17
Epha4	0.39	0.13	0.65	4.92	2.30	*1.09E-20*	2.68E-18
6330403K07Rik	0.35	0.12	0.58	4.89	2.29	*4.30E-18*	9.04E-16
Insm1	0.30	0.10	0.50	4.88	2.29	*5.62E-16*	9.75E-14
Zfp462	0.46	0.16	0.76	4.87	2.28	*7.83E-24*	2.28E-21
Pfn2	0.44	0.15	0.74	4.82	2.27	*1.60E-22*	4.33E-20
Rnd2	0.32	0.11	0.53	4.82	2.27	*4.73E-17*	8.99E-15
Mllt11	0.38	0.13	0.62	4.77	2.25	*6.73E-19*	1.47E-16
Neurog2	0.30	0.10	0.50	4.74	2.24	*2.70E-15*	4.38E-13
Kif1a	0.30	0.11	0.49	4.59	2.20	*1.25E-14*	1.88E-12
Gng3	0.31	0.11	0.51	4.59	2.20	*4.76E-15*	7.43E-13
Mpped2	0.35	0.13	0.57	4.57	2.19	*2.17E-17*	4.22E-15
Pantr1	0.31	0.11	0.51	4.55	2.19	*1.74E-15*	2.88E-13
Gm3764	0.30	0.11	0.49	4.55	2.19	*1.25E-14*	1.88E-12
Tmeff1	0.32	0.12	0.52	4.54	2.18	*6.64E-16*	1.14E-13
Dpysl5	0.29	0.11	0.47	4.49	2.17	*2.03E-14*	2.98E-12
Tbr1	0.28	0.10	0.46	4.48	2.16	*8.96E-14*	1.24E-11
Ppp2r2b	0.28	0.10	0.46	4.45	2.15	*8.96E-14*	1.24E-11
Neurod1	0.27	0.10	0.45	4.38	2.13	*1.46E-13*	1.97E-11

Plxna2	0.37	0.14	0.60	4.34	2.12	*1.47E-17*	2.92E-15
Cnih2	0.30	0.11	0.49	4.32	2.11	*3.49E-14*	4.99E-12
Bex2	0.28	0.11	0.45	4.31	2.11	*1.48E-13*	2.00E-11
Pou3f2	0.30	0.11	0.48	4.31	2.11	*3.46E-14*	4.97E-12
Zbtb18	0.43	0.17	0.70	4.22	2.08	*3.14E-19*	7.03E-17
Myt1l	0.26	0.10	0.42	4.22	2.08	*2.77E-12*	3.42E-10
Mir124-2hg	0.27	0.10	0.43	4.20	2.07	*1.70E-12*	2.14E-10
Camta1	0.31	0.12	0.50	4.20	2.07	*1.32E-14*	1.97E-12
Sox5	0.32	0.12	0.51	4.14	2.05	*3.46E-14*	4.97E-12
Mex3a	0.58	0.23	0.93	4.04	2.02	*1.15E-23*	3.31E-21
Ndn	0.50	0.20	0.80	4.04	2.01	*9.95E-21*	2.47E-18
Setbp1	0.38	0.15	0.61	4.02	2.01	*2.63E-16*	4.66E-14
Ttc3	1.47	0.59	2.36	4.02	2.01	*4.41E-58*	5.01E-55
Stmn3	0.27	0.11	0.43	4.02	2.01	*4.37E-12*	5.29E-10
Ptprz1	0.29	0.12	0.46	4.00	2.00	*2.52E-12*	3.14E-10
Srgap3	0.27	0.11	0.43	3.99	2.00	*1.76E-11*	2.06E-09
Sox4	1.52	0.61	2.43	3.98	1.99	*3.68E-59*	4.32E-56
Cxadr	0.25	0.10	0.41	3.96	1.99	*7.55E-11*	8.10E-09
Hdgfrp3	0.33	0.13	0.52	3.92	1.97	*1.33E-13*	1.80E-11
Stmn4	0.26	0.10	0.41	3.91	1.97	*4.64E-11*	5.13E-09
Fezf2	0.26	0.11	0.42	3.91	1.97	*2.86E-11*	3.25E-09
Rufy3	0.37	0.15	0.58	3.90	1.96	*4.33E-15*	6.86E-13
Ptbp2	0.37	0.15	0.59	3.89	1.96	*1.71E-15*	2.85E-13
Epha5	0.34	0.14	0.55	3.88	1.95	*1.92E-14*	2.83E-12
2610203C20Rik	0.26	0.11	0.41	3.85	1.94	*7.20E-11*	7.80E-09
Gnao1	0.32	0.13	0.51	3.83	1.94	*3.34E-13*	4.41E-11

Table S4

Related to Figure 2

List of genes most strongly enriched in mural cell clusters of the scRNA-seq dataset.

Gene	Base Mean	Base MeanA	Base MeanB	fold-enrichment	log2 [enrichment]	*p* value	FDR
Rgs5	7.38	0.28	14.49	52.50	5.71	0.00E+00	0.00E+00
Mmp9	2.34	0.14	4.54	33.12	5.05	0.00E+00	0.00E+00
Vtn	2.02	0.15	3.90	26.66	4.74	0.00E+00	0.00E+00
Igf2	7.50	0.65	14.34	21.97	4.46	0.00E+00	0.00E+00
Atp1a2	1.61	0.15	3.07	20.34	4.35	2.63E-222	1.49E-218
Abcc9	1.54	0.15	2.93	19.04	4.25	1.27E-207	6.16E-204
Cd248	0.85	0.11	1.58	13.84	3.79	2.42E-103	6.35E-100
Cald1	2.30	0.32	4.28	13.38	3.74	6.53E-255	4.45E-251
Kcnj8	0.77	0.11	1.43	12.87	3.69	1.50E-91	2.83E-88
Pcdh18	1.06	0.15	1.97	12.86	3.69	1.26E-125	4.28E-122
Myl9	0.88	0.13	1.64	12.33	3.62	7.53E-103	1.83E-99
Cspg4	0.75	0.11	1.38	12.29	3.62	1.74E-86	2.58E-83
Pdgfrb	0.68	0.11	1.25	11.75	3.55	1.89E-77	2.39E-74
Mylk	0.82	0.14	1.49	10.29	3.36	5.20E-87	8.05E-84
Heyl	0.58	0.11	1.05	9.67	3.27	4.27E-60	3.54E-57
Gjc1	0.96	0.19	1.74	9.29	3.22	4.00E-97	8.01E-94
Ednra	0.56	0.11	1.01	9.05	3.18	1.31E-56	9.90E-54
Dlk1	0.74	0.15	1.32	9.04	3.18	6.24E-74	6.85E-71
Col3a1	0.56	0.11	1.00	8.86	3.15	3.04E-55	2.25E-52
Rgs4	0.65	0.13	1.17	8.79	3.14	3.81E-64	3.51E-61
Ndufa4l2	0.49	0.11	0.86	7.97	2.99	3.31E-45	1.94E-42
Notch3	0.54	0.12	0.97	7.90	2.98	9.65E-51	6.57E-48
F2r	0.87	0.21	1.53	7.32	2.87	2.35E-75	2.76E-72
Fstl1	1.46	0.35	2.57	7.29	2.87	3.29E-125	1.02E-121
Col1a2	0.43	0.11	0.76	7.08	2.82	4.32E-38	2.02E-35
Gucy1b3	0.45	0.11	0.79	7.05	2.82	1.54E-39	7.59E-37
Gucy1a3	0.45	0.11	0.79	6.95	2.80	1.08E-38	5.11E-36
Atp1b2	0.51	0.14	0.89	6.54	2.71	4.68E-42	2.53E-39
40787	1.33	0.36	2.30	6.46	2.69	1.11E-105	3.16E-102
Ngfr	0.43	0.12	0.73	6.25	2.64	5.67E-34	2.33E-31
S1pr3	0.38	0.10	0.65	6.22	2.64	5.22E-30	1.76E-27
Zic1	0.91	0.26	1.57	6.12	2.61	4.22E-70	4.11E-67
Meg3	1.97	0.57	3.37	5.95	2.57	1.55E-141	5.86E-138
Sdc2	0.38	0.11	0.65	5.77	2.53	2.08E-28	6.80E-26
Mfge8	0.66	0.20	1.12	5.63	2.49	1.32E-47	8.50E-45
Farp1	0.45	0.14	0.76	5.54	2.47	1.38E-32	5.15E-30
Axl	0.48	0.15	0.81	5.36	2.42	1.52E-33	6.10E-31
Gm13889	0.46	0.15	0.78	5.32	2.41	3.73E-32	1.35E-29
Gper1	0.33	0.11	0.54	4.98	2.31	6.98E-22	1.77E-19
Uaca	0.51	0.17	0.84	4.93	2.30	5.82E-33	2.20E-30
S100a11	0.57	0.19	0.95	4.92	2.30	4.23E-37	1.89E-34
Tbxa2r	0.35	0.12	0.57	4.87	2.28	1.75E-22	4.59E-20
Mmp11	0.30	0.10	0.50	4.85	2.28	3.91E-20	9.30E-18
Pde8b	0.32	0.11	0.54	4.84	2.27	5.11E-21	1.27E-18
Lhfp	0.36	0.12	0.59	4.81	2.27	6.04E-23	1.60E-20
Foxd1	0.30	0.10	0.49	4.72	2.24	2.82E-19	6.27E-17
38231	0.45	0.16	0.73	4.67	2.22	1.11E-27	3.48E-25
Pde5a	0.37	0.13	0.60	4.54	2.18	1.18E-22	3.12E-20
Tppp3	0.36	0.13	0.58	4.48	2.16	5.71E-22	1.46E-19
Hspa1a|Hspa1b	0.89	0.33	1.45	4.42	2.14	1.01E-50	6.73E-48
Bgn	1.44	0.54	2.35	4.32	2.11	3.44E-79	4.51E-76
39326	1.39	0.52	2.26	4.31	2.11	6.70E-76	8.15E-73
Akap12	0.61	0.23	1.00	4.31	2.11	2.67E-34	1.11E-31
Rian	0.50	0.19	0.81	4.25	2.09	7.17E-28	2.30E-25
Trpc3	0.28	0.11	0.46	4.24	2.08	1.95E-16	3.71E-14
Plce1	0.29	0.11	0.47	4.22	2.08	4.05E-17	8.01E-15
Mcam	0.82	0.31	1.32	4.22	2.08	1.35E-44	7.64E-42
Gnas	0.85	0.33	1.38	4.20	2.07	1.40E-45	8.34E-43
Phlda1	0.75	0.29	1.20	4.18	2.06	5.02E-40	2.59E-37
Gadd45b	0.43	0.17	0.69	4.11	2.04	5.73E-23	1.54E-20
Tbx2	0.29	0.11	0.46	4.10	2.04	3.97E-16	7.30E-14
Lamb1	0.90	0.36	1.44	4.06	2.02	1.83E-46	1.16E-43
Fos	1.75	0.69	2.81	4.06	2.02	1.83E-88	2.96E-85
Arhgap42	0.31	0.13	0.50	3.99	2.00	3.38E-17	6.73E-15
Arhgef17	0.30	0.12	0.47	3.96	1.99	1.33E-15	2.40E-13
Gprc5c	0.25	0.10	0.40	3.96	1.98	1.10E-13	1.75E-11
Nid1	1.74	0.71	2.76	3.92	1.97	6.49E-84	9.21E-81
Ifitm3	0.35	0.14	0.56	3.87	1.95	3.05E-18	6.41E-16
Ptn	0.64	0.26	1.02	3.87	1.95	1.22E-31	4.32E-29
Higd1b	0.25	0.10	0.40	3.85	1.95	3.14E-13	4.82E-11
Eva1b	0.51	0.21	0.81	3.82	1.93	6.24E-25	1.82E-22
Tns1	0.42	0.17	0.66	3.80	1.93	1.89E-20	4.55E-18
Epb41l1	0.31	0.13	0.49	3.77	1.92	4.84E-16	8.87E-14
Lgals1	0.35	0.15	0.55	3.76	1.91	1.37E-17	2.82E-15

Cdh11	0.39	0.17	0.62	3.73	1.90	7.87E-19	1.70E-16
Tnfrsf21	0.48	0.20	0.76	3.72	1.90	2.12E-23	5.87E-21
Igf2r	0.51	0.22	0.80	3.66	1.87	5.83E-24	1.65E-21
Ebf1	0.79	0.34	1.24	3.65	1.87	3.94E-36	1.72E-33
Gstk1	0.25	0.11	0.40	3.63	1.86	6.71E-13	1.01E-10
Afap1l2	0.25	0.11	0.40	3.63	1.86	2.12E-12	3.03E-10
Atp7a	0.45	0.20	0.71	3.58	1.84	7.43E-21	1.83E-18
Pdlim2	0.26	0.11	0.40	3.58	1.84	4.38E-12	6.18E-10
Spry4	0.26	0.11	0.40	3.55	1.83	2.30E-12	3.27E-10
Adap2	0.33	0.15	0.52	3.53	1.82	2.38E-15	4.22E-13
Sema6d	1.00	0.44	1.56	3.53	1.82	2.05E-43	1.14E-40
Dmd	0.26	0.11	0.41	3.53	1.82	1.92E-12	2.77E-10
Col5a1	0.25	0.11	0.39	3.51	1.81	1.00E-11	1.36E-09
Oaz2	0.71	0.31	1.10	3.51	1.81	8.33E-31	2.92E-28
Timp3	0.45	0.20	0.70	3.49	1.80	1.63E-20	3.99E-18
Sfrp2	0.25	0.11	0.38	3.46	1.79	1.00E-11	1.36E-09
Zfp703	0.44	0.20	0.68	3.46	1.79	1.57E-19	3.56E-17
Pid1	0.35	0.16	0.54	3.45	1.79	2.50E-15	4.40E-13
Hic1	0.29	0.13	0.44	3.45	1.79	6.07E-13	9.22E-11
Tlr12	0.27	0.12	0.42	3.45	1.79	1.43E-12	2.09E-10
Tbx18	0.23	0.10	0.35	3.41	1.77	2.24E-10	2.66E-08
Car4	0.23	0.10	0.36	3.41	1.77	6.54E-11	8.15E-09
Rasl12	0.23	0.11	0.36	3.41	1.77	9.84E-11	1.19E-08
Lamc3	0.23	0.11	0.36	3.39	1.76	9.84E-11	1.19E-08
Slc19a1	0.24	0.11	0.37	3.34	1.74	7.09E-11	8.81E-09
Myh9	0.76	0.35	1.16	3.31	1.73	1.87E-30	6.48E-28

Table S5

Related to Figure 2

List of genes most strongly enriched in endothelial clusters of the scRNA-seq dataset.

Gene	Base Mean	Base MeanA	Base MeanB	fold-enrichment	log2 [enrichment]	*p* value	FDR
Cldn5	1.73	0.12	3.35	28.42	4.83	5.81E-260	9.88E-256
Kdr	1.73	0.13	3.32	25.30	4.66	6.89E-252	7.81E-248
Cd93	1.62	0.13	3.11	24.54	4.62	1.48E-234	1.26E-230
Spock2	1.45	0.16	2.74	17.58	4.14	1.13E-189	5.50E-186
Flt1	1.50	0.16	2.83	17.28	4.11	1.28E-196	7.26E-193
Slc2a1	1.52	0.19	2.84	15.07	3.91	2.55E-189	1.08E-185
Vwa1	0.84	0.12	1.57	12.92	3.69	3.23E-99	7.33E-96
Slc7a5	1.38	0.21	2.55	11.86	3.57	9.76E-157	3.32E-153
Slc38a5	0.67	0.11	1.24	11.15	3.48	5.20E-75	7.08E-72
Ptprb	0.71	0.12	1.29	10.78	3.43	5.98E-77	8.85E-74
Egfl7	0.76	0.13	1.38	10.76	3.43	2.26E-82	4.05E-79
Slc7a1	1.87	0.32	3.42	10.63	3.41	2.80E-199	1.91E-195
Tie1	0.71	0.13	1.29	9.80	3.29	1.78E-74	2.25E-71
Pecam1	0.63	0.12	1.15	9.76	3.29	6.48E-66	7.12E-63
Mfsd2a	0.54	0.11	0.96	8.68	3.12	8.71E-53	6.89E-50
Mmrn2	0.55	0.12	0.98	8.49	3.09	5.38E-53	4.36E-50
Slc38a3	0.51	0.11	0.90	8.34	3.06	2.50E-48	1.70E-45
Htra3	0.51	0.11	0.91	7.95	2.99	1.18E-47	7.90E-45
Cd34	0.61	0.14	1.09	7.95	2.99	1.32E-56	1.25E-53
Cdh5	0.44	0.11	0.78	7.26	2.86	1.83E-39	1.01E-36
Nos3	0.41	0.10	0.72	6.84	2.77	1.04E-34	4.65E-32
Adgrl4	0.43	0.11	0.74	6.77	2.76	8.60E-36	4.01E-33
Col18a1	0.44	0.12	0.77	6.64	2.73	1.92E-36	9.61E-34
AU021092	0.39	0.10	0.67	6.55	2.71	1.87E-32	7.65E-30
Slco1c1	0.40	0.11	0.70	6.49	2.70	4.12E-33	1.73E-30
Itm2a	0.99	0.27	1.72	6.30	2.65	9.91E-77	1.41E-73
Slc1a4	0.59	0.16	1.01	6.17	2.62	9.36E-46	6.01E-43
Zic3	0.52	0.15	0.89	6.15	2.62	1.19E-40	6.66E-38
Tspan18	0.50	0.14	0.85	5.85	2.55	3.01E-37	1.55E-34
Lmo2	0.60	0.18	1.02	5.79	2.53	4.09E-44	2.44E-41
Tfrc	0.67	0.20	1.15	5.77	2.53	1.45E-49	1.05E-46
Ramp2	0.68	0.20	1.17	5.74	2.52	2.80E-50	2.07E-47
Rasip1	0.41	0.12	0.70	5.72	2.52	2.68E-30	9.69E-28
Lsr	0.38	0.11	0.64	5.69	2.51	9.28E-28	3.07E-25
Slc40a1	0.50	0.15	0.85	5.59	2.48	6.17E-36	2.96E-33
Myo10	0.69	0.21	1.18	5.56	2.48	1.92E-49	1.36E-46
Eogt	0.40	0.12	0.67	5.54	2.47	1.11E-28	3.86E-26
Limch1	0.45	0.14	0.76	5.49	2.46	1.07E-31	4.08E-29
Sox18	0.33	0.10	0.56	5.48	2.45	6.61E-24	1.89E-21
Plxnd1	0.43	0.14	0.73	5.36	2.42	1.31E-30	4.84E-28
Mpzl1	0.55	0.17	0.93	5.33	2.41	9.54E-38	5.07E-35
Slc39a8	0.40	0.13	0.67	5.26	2.40	9.99E-28	3.27E-25
Adgrf5	0.50	0.16	0.83	5.26	2.40	8.70E-34	3.80E-31
Aplnr	0.32	0.10	0.54	5.23	2.39	2.82E-22	7.26E-20
Slc6a6	1.05	0.35	1.76	5.08	2.34	7.13E-68	8.37E-65
Slc38a2	2.31	0.77	3.86	5.03	2.33	1.32E-134	4.07E-131
Abcb1a	0.31	0.10	0.51	4.92	2.30	4.16E-20	9.83E-18
Prnp	0.49	0.17	0.81	4.92	2.30	2.15E-31	8.06E-29
Tsc22d1	2.10	0.71	3.48	4.87	2.29	6.04E-123	1.58E-119
Slc39a10	0.61	0.21	1.01	4.72	2.24	6.00E-38	3.24E-35
Apod	0.90	0.32	1.49	4.68	2.23	2.84E-54	2.42E-51
Lrp8	0.38	0.14	0.62	4.55	2.18	8.99E-23	2.39E-20
Thsd1	0.30	0.11	0.49	4.54	2.18	4.32E-18	9.20E-16
Robo4	0.28	0.10	0.46	4.53	2.18	1.35E-17	2.72E-15
Ccnjl	0.38	0.14	0.63	4.51	2.17	3.75E-23	1.03E-20
Enpp2	0.29	0.10	0.47	4.50	2.17	2.17E-17	4.30E-15
Apln	0.29	0.10	0.47	4.48	2.16	2.17E-17	4.30E-15
Plvap	0.30	0.11	0.50	4.47	2.16	8.61E-19	1.91E-16
Bsg	0.97	0.36	1.58	4.43	2.15	1.36E-54	1.18E-51
Sox17	0.28	0.10	0.45	4.42	2.14	5.71E-17	1.09E-14
Nampt	0.49	0.18	0.81	4.40	2.14	1.89E-28	6.49E-26
Palmd	0.31	0.11	0.50	4.39	2.13	2.57E-18	5.56E-16
Eng	0.49	0.18	0.80	4.37	2.13	3.94E-28	1.31E-25
Kank3	0.30	0.11	0.49	4.36	2.12	4.87E-18	1.03E-15
Tek	0.40	0.15	0.65	4.34	2.12	1.16E-22	3.05E-20
Pde8a	0.33	0.12	0.53	4.33	2.12	8.93E-19	1.97E-16
Ctla2a	0.30	0.11	0.48	4.32	2.11	3.97E-17	7.59E-15
Mllt4	0.57	0.22	0.93	4.27	2.10	1.39E-31	5.24E-29
Podxl	0.31	0.12	0.50	4.27	2.10	7.15E-18	1.48E-15
Dock9	0.31	0.12	0.50	4.26	2.09	7.15E-18	1.48E-15
Ablim1	0.58	0.22	0.94	4.24	2.08	2.88E-32	1.17E-29
Gja1	0.28	0.11	0.45	4.19	2.07	4.57E-16	8.14E-14
Ece1	0.31	0.12	0.49	4.13	2.05	7.03E-17	1.32E-14

Myh10	0.85	0.33	1.37	4.12	2.04	1.04E-44	6.43E-42
Erg	0.26	0.10	0.41	4.10	2.03	2.53E-14	4.01E-12
Esam	0.69	0.27	1.11	4.06	2.02	5.78E-36	2.81E-33
Gm28045|Prnd	0.25	0.10	0.40	4.02	2.01	1.47E-13	2.18E-11
Mecom	0.27	0.11	0.43	4.01	2.00	1.35E-14	2.19E-12
Cyyr1	0.27	0.11	0.43	4.00	2.00	2.07E-14	3.32E-12
Sh3bp5	0.42	0.17	0.68	4.00	2.00	6.14E-22	1.57E-19
Adm	0.31	0.12	0.49	3.99	2.00	2.09E-16	3.83E-14
Kcp	0.34	0.14	0.54	3.98	1.99	3.82E-18	8.23E-16
Cdc42ep3	0.31	0.13	0.49	3.94	1.98	4.64E-16	8.22E-14
Sema6a	0.65	0.26	1.04	3.93	1.98	2.95E-32	1.18E-29
Fli1	0.40	0.16	0.64	3.89	1.96	1.58E-20	3.81E-18
Ccdc141	0.39	0.16	0.62	3.87	1.95	7.16E-20	1.67E-17
Ptprg	0.48	0.20	0.77	3.87	1.95	2.69E-24	7.91E-22
Plk2	0.34	0.14	0.54	3.82	1.93	2.88E-17	5.57E-15
Ecscr	0.38	0.16	0.60	3.82	1.93	2.14E-19	4.93E-17
Dok4	0.27	0.11	0.43	3.80	1.93	4.26E-14	6.65E-12
Csrp2	0.47	0.20	0.74	3.76	1.91	1.11E-22	2.92E-20
Lef1	0.29	0.12	0.46	3.76	1.91	1.61E-14	2.58E-12
Fn1	1.23	0.52	1.95	3.75	1.91	4.92E-57	4.78E-54
Efna1	0.25	0.11	0.40	3.72	1.90	1.61E-12	2.19E-10
Slc3a2	1.80	0.77	2.83	3.69	1.88	1.96E-80	3.18E-77
Sox7	0.24	0.10	0.38	3.68	1.88	8.83E-12	1.15E-09
Adamtsl2	0.23	0.10	0.37	3.67	1.88	1.12E-11	1.43E-09
Abca1	0.93	0.40	1.46	3.67	1.88	3.11E-42	1.76E-39
Tdrp	0.24	0.10	0.37	3.62	1.86	6.17E-12	8.11E-10
Apcdd1	0.28	0.12	0.44	3.60	1.85	1.87E-13	2.67E-11

Table S6

Related to Figure 2

List of genes most strongly enriched in the microglia cluster of the scRNA-seq dataset.

Gene	Base Mean	Base MeanA	Base MeanB	fold-enrichment	log2 [enrichment]	*p* value	FDR
C1qb	3.56	0.20	6.93	34.99	5.13	0.00E+00	0.00E+00
Ctss	2.87	0.17	5.57	33.32	5.06	0.00E+00	0.00E+00
C1qc	2.13	0.14	4.13	29.80	4.90	0.00E+00	0.00E+00
Csf1r	2.28	0.15	4.40	28.70	4.84	0.00E+00	0.00E+00
Ly86	1.76	0.12	3.39	27.79	4.80	7.45E-222	2.54E-218
Hexb	2.26	0.18	4.34	24.59	4.62	0.00E+00	0.00E+00
Ccl4	1.33	0.12	2.53	21.13	4.40	1.72E-155	3.08E-152
Cx3cr1	1.38	0.12	2.64	21.12	4.40	4.75E-161	1.01E-157
P2ry12	1.25	0.11	2.38	20.79	4.38	8.41E-145	1.30E-141
Fcer1g	1.38	0.13	2.62	20.05	4.33	9.43E-158	1.78E-154
Fcrls	1.24	0.12	2.35	19.62	4.29	1.46E-141	2.16E-138
Apoe	7.48	0.76	14.21	18.62	4.22	0.00E+00	0.00E+00
Mpeg1	1.15	0.12	2.18	18.48	4.21	5.02E-129	6.84E-126
C3ar1	1.18	0.12	2.24	18.02	4.17	7.47E-131	1.06E-127
Ctsd	2.35	0.25	4.45	17.67	4.14	0.00E+00	0.00E+00
Ctsb	4.30	0.47	8.13	17.24	4.11	0.00E+00	0.00E+00
Lgmn	1.52	0.17	2.88	17.05	4.09	5.47E-165	1.24E-161
C1qa	0.99	0.11	1.87	16.55	4.05	1.13E-106	1.33E-103
Tyrobp	0.87	0.11	1.64	15.01	3.91	1.02E-90	1.06E-87
Cd53	0.96	0.14	1.78	12.92	3.69	6.42E-94	6.83E-91
Laptm5	1.96	0.30	3.63	11.96	3.58	3.23E-179	7.84E-176
Kctd12	0.95	0.16	1.73	11.17	3.48	6.03E-86	5.86E-83
Atf3	1.46	0.26	2.66	10.32	3.37	4.75E-126	6.22E-123
Lyz1|Lyz2	0.64	0.11	1.16	10.24	3.36	3.82E-56	2.50E-53
Fcgr3	0.62	0.11	1.13	10.07	3.33	2.52E-54	1.56E-51
Hpgds	0.67	0.12	1.22	10.06	3.33	9.10E-58	6.20E-55
Grn	1.92	0.35	3.48	9.99	3.32	1.08E-159	2.16E-156
Plek	0.61	0.11	1.10	9.71	3.28	1.21E-51	7.00E-49
Ccl3	0.71	0.14	1.29	9.40	3.23	2.24E-59	1.66E-56
Cd83	0.66	0.13	1.20	9.37	3.23	6.01E-55	3.86E-52
Coro1a	0.58	0.11	1.06	9.25	3.21	6.79E-48	3.67E-45
Ctsl	1.49	0.30	2.68	8.97	3.16	3.02E-118	3.80E-115
Aif1	0.54	0.11	0.97	8.91	3.15	1.28E-43	6.04E-41
Siglech	0.51	0.10	0.91	8.76	3.13	5.42E-41	2.22E-38
Cst3	2.89	0.60	5.17	8.58	3.10	7.51E-194	2.32E-190
Ptgs1	0.53	0.11	0.95	8.49	3.09	3.89E-42	1.70E-39
Sirpa	0.65	0.14	1.16	8.47	3.08	3.74E-51	2.12E-48
Lpl	0.64	0.14	1.14	7.90	2.98	1.65E-47	8.77E-45
Cd52	0.45	0.10	0.79	7.65	2.94	1.77E-33	5.90E-31
Bin2	0.52	0.12	0.91	7.55	2.92	1.28E-37	4.85E-35
Spp1	0.43	0.10	0.75	7.37	2.88	3.26E-31	1.00E-28
Itgam	0.46	0.11	0.80	7.27	2.86	6.08E-33	2.01E-30
Olfml3	0.59	0.14	1.03	7.26	2.86	6.20E-42	2.67E-39
Trem2	0.43	0.10	0.75	7.23	2.85	7.25E-31	2.18E-28
Ly6e	0.76	0.19	1.34	7.23	2.85	5.09E-54	3.04E-51
Ncf1	0.43	0.11	0.76	7.02	2.81	2.29E-30	6.79E-28
P2ry13	0.43	0.11	0.76	6.90	2.79	2.29E-30	6.79E-28
Cfh	0.50	0.13	0.87	6.88	2.78	2.37E-34	8.16E-32
B2m	2.47	0.63	4.31	6.82	2.77	4.58E-148	7.80E-145
Pld4	0.41	0.11	0.72	6.77	2.76	1.78E-28	5.08E-26
Adgre1	0.40	0.10	0.70	6.76	2.76	8.69E-28	2.37E-25
Evi2a	0.42	0.11	0.72	6.67	2.74	7.84E-28	2.15E-25
Unc93b1	0.49	0.13	0.85	6.64	2.73	1.09E-32	3.47E-30
Il1a	0.40	0.11	0.70	6.56	2.71	3.81E-27	1.01E-24
Fyb	0.40	0.11	0.70	6.55	2.71	1.21E-26	3.16E-24
Psap	1.14	0.31	1.97	6.45	2.69	1.40E-72	1.17E-69
Apbb1ip	0.38	0.10	0.65	6.42	2.68	4.36E-25	1.02E-22
Cd180	0.36	0.10	0.63	6.17	2.63	1.34E-23	2.92E-21
Cd33	0.40	0.11	0.70	6.06	2.60	1.98E-25	4.78E-23
Il6ra	0.40	0.12	0.69	5.99	2.58	1.98E-25	4.78E-23
Cyth4	0.39	0.11	0.67	5.99	2.58	1.47E-24	3.37E-22
Ctsz	0.85	0.25	1.45	5.93	2.57	9.97E-51	5.48E-48
Tgfbr1	0.73	0.21	1.25	5.88	2.56	4.12E-43	1.92E-40
Pde3b	0.43	0.12	0.73	5.87	2.55	6.47E-26	1.61E-23
Nckap1l	0.36	0.11	0.61	5.79	2.53	4.86E-22	9.62E-20
Gpr34	0.35	0.10	0.60	5.78	2.53	7.63E-22	1.50E-19
Rnase4	0.80	0.24	1.36	5.77	2.53	2.12E-46	1.08E-43
Rac2	0.37	0.11	0.63	5.72	2.52	3.76E-22	7.49E-20
Arhgap25	0.44	0.13	0.74	5.61	2.49	9.54E-26	2.34E-23
Serinc3	2.18	0.67	3.69	5.51	2.46	4.48E-113	5.45E-110
Ctsc	0.57	0.18	0.96	5.34	2.42	1.86E-31	5.75E-29
Man2b1	0.48	0.15	0.80	5.34	2.42	1.33E-26	3.47E-24
Pou2f2	0.46	0.15	0.78	5.33	2.41	5.32E-26	1.33E-23

Myo1f	0.33	0.11	0.56	5.29	2.40	9.81E-19	1.63E-16
Tpd52	0.43	0.14	0.73	5.23	2.39	5.22E-24	1.16E-21
Ptpn6	0.40	0.13	0.68	5.21	2.38	1.53E-22	3.18E-20
Lpcat2	0.32	0.10	0.54	5.20	2.38	2.84E-18	4.65E-16
Ptprc	0.34	0.11	0.57	5.19	2.38	3.87E-19	6.55E-17
Maf	0.68	0.22	1.14	5.17	2.37	7.56E-37	2.77E-34
Selplg	0.33	0.11	0.55	5.17	2.37	2.84E-18	4.65E-16
Gusb	0.66	0.22	1.10	5.07	2.34	2.30E-34	7.98E-32
Ncf2	0.38	0.13	0.64	5.02	2.33	1.65E-20	3.04E-18
Rgs10	0.39	0.13	0.65	5.00	2.32	3.64E-21	6.85E-19
Mt1	0.43	0.14	0.72	4.97	2.31	2.42E-22	4.87E-20
Cd9	0.49	0.16	0.81	4.96	2.31	2.80E-25	6.63E-23
Abhd12	0.46	0.16	0.77	4.86	2.28	9.00E-24	1.98E-21
Lgals9	0.46	0.16	0.77	4.84	2.28	9.00E-24	1.98E-21
Trib1	0.59	0.20	0.97	4.82	2.27	3.73E-29	1.07E-26
Cd84	0.32	0.11	0.53	4.76	2.25	3.47E-16	5.14E-14
Arpc1b	1.23	0.43	2.03	4.74	2.25	5.23E-59	3.71E-56
Neat1	0.42	0.15	0.70	4.73	2.24	2.04E-21	3.87E-19
Plin2	0.51	0.18	0.83	4.62	2.21	1.05E-24	2.42E-22
Ifrd1	0.69	0.25	1.14	4.62	2.21	8.82E-33	2.89E-30
Vsir	0.36	0.13	0.59	4.59	2.20	7.76E-18	1.25E-15
Runx1	0.32	0.11	0.52	4.57	2.19	1.00E-15	1.44E-13
Parvg	0.30	0.11	0.49	4.55	2.19	6.75E-15	9.26E-13
Ifi30	0.36	0.13	0.59	4.55	2.18	1.31E-17	2.09E-15
Hexa	0.46	0.17	0.75	4.52	2.18	1.57E-21	3.02E-19
Sgpl1	0.44	0.16	0.71	4.51	2.17	1.05E-20	1.94E-18
Arl4c	0.40	0.15	0.66	4.49	2.17	4.89E-19	8.21E-17

Table S7

Related to Figure 3

Top 50 enriched genes in the EMBRACE-isolated neural population (CD45^{neg}, CD41^{neg}, CD11b^{neg}, PECAM1^{neg}, CD102^{neg}, PDGFRβ^{neg}).

Gene	baseMean	log2FC	p value	FDR
Gm42418	4045.63	15.89	3.35E-88	6.93E-85
Ptprn2	294.82	11.60	6.49E-55	3.42E-52
Slc32a1	220.18	11.50	3.14E-55	1.72E-52
Gm8203	177.07	11.48	1.41E-49	5.10E-47
Xkr7	169.99	11.43	2.44E-50	9.79E-48
Ecel1	166.55	11.40	3.71E-49	1.31E-46
Pdcd2	132.45	11.08	1.11E-46	3.44E-44
Myo16	194.24	11.02	5.57E-53	2.63E-50
Nalcn	119.54	10.93	1.08E-45	3.19E-43
Shisa6	106.85	10.77	1.68E-43	4.30E-41
Gpr26	129.34	10.74	4.48E-47	1.43E-44
Uncx	157.31	10.69	1.16E-43	3.02E-41
Gjd2	90.05	10.52	4.59E-41	9.78E-39
Tmem28	106.58	10.46	7.67E-43	1.89E-40
Fam189a1	105.19	10.45	1.83E-44	4.97E-42
Rtn4r	85.74	10.44	6.58E-38	1.14E-35
Reln	1791.73	10.40	6.61E-18	2.21E-16
Grm4	79.77	10.35	1.27E-39	2.42E-37
Islr2	4212.44	10.33	2.29E-31	2.54E-29
Lrrn2	270.23	10.25	3.59E-65	3.32E-62
Tmem59l	111.39	10.23	3.44E-45	9.72E-43
Kndc1	90.12	10.23	1.93E-42	4.52E-40
Erbb4	334.30	10.20	2.55E-63	2.21E-60
Gm12481	69.70	10.14	3.11E-35	4.52E-33
Miat	13098.89	10.09	2.87E-46	8.66E-44
Gm5812	64.73	10.05	8.99E-37	1.50E-34
Hrh3	76.46	10.00	4.83E-39	8.89E-37
Gabra3	141.92	10.00	5.50E-47	1.74E-44
Mmp24	430.49	9.99	1.46E-11	2.30E-10
Galnt14	61.39	9.98	5.85E-36	9.08E-34
Zim1	94.94	9.96	2.45E-35	3.60E-33
Sorcs3	75.21	9.95	3.73E-36	5.96E-34
Magel2	868.39	9.94	2.44E-27	2.01E-25
Frmpd3	91.12	9.92	1.82E-37	3.10E-35
Epha8	87.93	9.90	2.04E-41	4.42E-39
Chrna3	56.23	9.84	1.61E-33	2.18E-31
Kcnf1	66.83	9.80	1.81E-38	3.25E-36
Disp2	604.76	9.78	9.20E-14	1.93E-12
Thsd7b	65.77	9.76	4.70E-36	7.38E-34
Gm996	63.80	9.74	2.76E-38	4.85E-36
Gm27032	51.06	9.71	8.27E-34	1.13E-31
Scg2	118.04	9.69	5.71E-18	1.92E-16
Tunar	47.92	9.62	3.46E-32	4.22E-30
Tenm1	73.28	9.57	1.08E-30	1.12E-28
Zfhx2os	56.80	9.57	2.86E-36	4.63E-34
Lgi2	46.12	9.57	3.54E-32	4.31E-30
Rgs7	46.23	9.56	2.44E-30	2.46E-28
Adcyap1	44.62	9.51	2.42E-31	2.68E-29
Syde2	54.11	9.50	7.23E-36	1.12E-33
Cnpy1	357.67	9.38	2.14E-16	6.08E-15

Table S8

Related to Figure 3

Top 50 enriched genes in the EMBRACE-isolated mural cell population (PDGFRβ^{high}, PECAM1^{neg}, CD102^{neg}, CD45^{neg}, CD41^{neg}, CD11b^{neg}). Due to their high abundance in the list, genes with the Gm prefix were removed from this list.

Gene	baseMean	log2FC	p value	FDR
Kcne4	933.90	12.51	1.60E-38	4.74E-35
Cpa1	309.65	11.90	9.97E-44	4.72E-40
Ednra	5589.26	11.86	2.58E-43	1.02E-39
Atp13a5	616.11	10.83	3.05E-18	1.90E-15
Enpep	222.42	10.58	1.57E-47	9.30E-44
Mmp9	11155.83	10.36	6.24E-20	5.09E-17
Alx1	156.94	10.25	2.15E-29	3.91E-26
Hcar1	87.23	10.23	1.15E-21	1.23E-18
Slc6a20a	254.82	10.03	2.22E-18	1.46E-15
4933431K23Rik	69.23	9.75	3.23E-26	4.78E-23
Rarres2	185.96	9.74	2.22E-35	5.83E-32
Vtn	12818.27	9.42	3.07E-15	1.17E-12
B830012L14Rik	214.23	9.27	1.53E-59	1.81E-55
Sod3	359.20	9.26	4.10E-31	8.82E-28
8030451A03Rik	84.20	9.05	3.30E-16	1.62E-13
Tbx18	406.60	8.69	2.12E-15	8.51E-13
Akr1c12	33.37	8.68	1.15E-11	2.12E-09
Vpreb3	30.63	8.61	4.61E-20	3.90E-17
Cd248	1895.31	8.12	2.09E-17	1.12E-14
Art3	1845.52	8.07	6.34E-24	7.89E-21
Ins2	22.41	8.03	5.27E-09	4.95E-07
Slc38a11	137.30	7.98	2.29E-19	1.75E-16
Postn	605.46	7.86	1.90E-15	7.76E-13
C1qtnf2	86.03	7.84	9.73E-16	4.19E-13
Myocd	30.12	7.81	6.25E-15	2.24E-12
1600015I10Rik	15.33	7.71	2.61E-10	3.52E-08
4921534H16Rik	16.11	7.61	2.30E-08	1.84E-06
Mylk4	86.57	7.57	2.05E-22	2.31E-19
Vstm4	663.01	7.55	4.08E-07	2.27E-05
Ndufa4l2	6822.54	7.55	2.89E-10	3.83E-08
Cspg4	2288.33	7.50	5.72E-12	1.14E-09
Krtdap	16.93	7.44	1.73E-06	7.67E-05
Kcnmb1	228.04	7.43	2.18E-08	1.75E-06
Myl9	8993.35	7.31	2.34E-24	3.08E-21
S1pr3	3632.06	7.28	5.15E-06	1.91E-04
S1pr3	3632.06	7.28	5.15E-06	0.000191
Npn2	12.38	7.25	1.29E-07	8.36E-06
2010003K11Rik	14.42	7.25	0.000873	0.010468
Col6a3	192.08	7.23	4.75E-07	2.56E-05
Rgs4	13901.09	7.21	9.88E-09	8.63E-07
Prss45	11.53	7.20	6.59E-08	4.64E-06
Bmp5	317.08	7.20	5.23E-07	2.78E-05
Lamc3	717.82	7.15	1.01E-07	6.79E-06
C1qtnf7	13.84	7.14	5.78E-09	5.34E-07
Gpr20	56.94	7.14	2.08E-08	1.69E-06
Tmc5	112.89	7.14	2.50E-07	1.50E-05
Des	125.96	7.12	2.75E-08	2.16E-06
Tnxb	106.90	7.11	6.84E-08	4.79E-06
Crygc	10.94	7.06	4.19E-07	2.32E-05
Higd1b	2370.20	7.00	8.42E-04	1.02E-02

Table S9

Related to Figure 3

Top 50 enriched genes in the EMBRACE-isolated endothelial cell population (PECAM1^{pos}, CD102^{pos}, CD45^{neg}, CD41^{neg}, CD11b^{neg}, PDGFRβ^{neg}).

Gene	baseMean	log2FC	p value	FDR
Myct1	675.89	12.81	1.24E-57	1.43E-53
Mogat2	510.34	12.66	4.39E-44	2.53E-40
Gimap4	332.25	12.38	8.88E-31	1.86E-27
Kcne3	362.22	12.19	3.43E-21	3.44E-18
Tbx1	339.36	11.70	4.42E-30	8.48E-27
Gm38197	377.99	11.45	1.09E-31	3.15E-28
Mall	212.27	11.43	4.55E-26	6.97E-23
Igsf5	101.69	10.67	2.16E-20	1.85E-17
Cfi	107.66	10.44	1.54E-33	5.05E-30
Nos3	1955.22	10.35	7.87E-34	3.02E-30
4930578C19Rik	85.41	9.91	8.92E-11	1.35E-08
A530016L24Rik	62.83	9.75	1.01E-12	2.38E-10
Gm37393	60.31	9.73	3.53E-13	9.23E-11
Gm694	47.25	9.73	7.35E-20	5.83E-17
Cldn5	14488.32	9.54	3.02E-09	3.26E-07
Rassf9	951.34	9.30	4.94E-13	1.23E-10
Gm12866	37.97	9.29	8.43E-15	2.85E-12
Rp1	108.20	9.28	1.92E-24	2.60E-21
Foxl2	237.78	9.27	1.61E-24	2.32E-21
Ces2b	53.19	9.22	4.80E-09	4.80E-07
BB365896	39.02	9.12	4.63E-17	2.42E-14
Clca2	51.76	9.02	1.13E-10	1.67E-08
Slc38a5	6137.35	9.00	4.45E-12	9.40E-10
Serpinb9b	78.97	8.98	2.89E-14	8.98E-12
9930038B18Rik	30.18	8.92	9.08E-13	2.18E-10
Mmrn1	1127.35	8.85	6.41E-15	2.23E-12
Gm38066	35.10	8.85	1.36E-09	1.62E-07
Psg17	39.10	8.83	1.70E-15	6.54E-13
Aplnr	4161.45	8.75	6.14E-08	4.53E-06
Gpihbp1	25.07	8.72	3.72E-13	9.51E-11
Unc45b	275.01	8.69	5.60E-11	8.99E-09
Vwa1	4369.62	8.68	3.22E-16	1.51E-13
Adgrl4	3021.45	8.58	3.63E-12	7.80E-10
Gpr143	32.49	8.44	8.44E-05	2.00E-03
Rhbdl2	262.17	8.40	1.98E-09	2.22E-07
Gm24283	36.06	8.40	1.53E-15	5.96E-13
Hapln1	232.78	8.30	8.39E-12	1.69E-09
Acsbg2	20.54	8.23	4.01E-09	4.18E-07
Myzap	406.72	8.20	2.11E-11	3.73E-09
Slco1c1	1776.55	8.20	4.04E-11	6.69E-09
Allc	22.61	8.15	3.07E-10	4.22E-08
Ushbp1	1659.27	8.13	9.69E-14	2.79E-11
Ptgis	730.64	8.03	9.58E-10	1.18E-07
Shbg	32.10	8.01	1.37E-09	1.63E-07
T	121.42	7.97	2.40E-10	3.37E-08
Prnd	159.22	7.92	3.75E-12	8.00E-10
Tecrl	18.10	7.86	2.62E-04	4.75E-03
Gm10258	22.95	7.84	8.03E-06	2.95E-04
Nos2	173.31	7.84	3.26E-10	4.47E-08
Foxl2os	56.12	7.84	2.04E-13	5.65E-11

Table S10

Related to Figure 3

Top 50 enriched genes in the EMBRACE-isolated microglia population (CD45^{medium}, CD11b^{pos}, PECAM1^{neg}, PDGFRβ^{neg}).

Gene	baseMean	log2FC	p value	FDR
Ctss	17584.46	17.68	2.32E-101	1.34E-97
Fcrls	15462.25	17.49	2.86E-99	1.32E-95
C1qc	13028.88	17.28	1.30E-101	9.95E-98
C1qb	17918.06	17.00	9.11E-130	1.05E-125
Fcgr3	7037.22	16.41	8.74E-90	3.36E-86
Ly86	4920.81	15.86	1.77E-77	3.72E-74
Trem2	4014.58	15.60	3.87E-78	8.92E-75
Adgre1	4387.59	15.24	3.40E-89	1.12E-85
C1qa	6834.11	15.16	1.14E-65	1.46E-62
Cd86	2583.89	15.14	1.60E-53	1.19E-50
Rgs1	1985.73	14.89	2.03E-61	2.34E-58
Cd84	2662.58	14.87	1.88E-55	1.61E-52
Siglech	3729.47	14.83	7.10E-76	1.28E-72
Tyrobp	4861.84	14.64	2.33E-41	1.12E-38
Rac2	3191.76	14.64	1.21E-73	1.86E-70
Spi1	3588.60	14.36	1.16E-78	2.98E-75
Clec5a	891.88	13.80	2.07E-54	1.64E-51
Ms4a6d	926.47	13.73	3.11E-44	1.59E-41
Ncf4	831.95	13.69	8.01E-53	5.77E-50
Tifab	957.65	13.57	7.27E-57	7.28E-54
Ms4a6b	924.84	13.51	2.27E-54	1.75E-51
Ms4a6c	760.16	13.51	2.13E-46	1.17E-43
Cd53	3796.61	13.49	5.30E-55	4.36E-52
Abcg3	828.87	13.35	1.77E-52	1.20E-49
Sash3	1171.07	13.34	2.82E-59	2.95E-56
Cd300c2	769.03	13.31	1.25E-59	1.37E-56
Ccr1	612.11	13.06	3.84E-35	1.38E-32
Bank1	644.68	13.05	1.27E-55	1.17E-52
Cx3cr1	13140.39	13.04	1.92E-39	8.06E-37
Tnfaip8l2	629.37	12.96	2.04E-49	1.24E-46
Ccl12	527.37	12.94	1.37E-38	5.53E-36
Clec7a	525.75	12.91	5.20E-37	2.00E-34
Itgam	1330.38	12.88	4.69E-66	6.36E-63
Nlrp3	826.51	12.85	1.76E-55	1.56E-52
Cd48	466.81	12.84	7.48E-43	3.67E-40
Casp1	706.43	12.82	1.63E-52	1.14E-49
Dock2	2034.59	12.81	3.76E-29	9.11E-27
Ptprc	4962.73	12.81	2.46E-37	9.62E-35
Ly9	405.13	12.70	4.00E-46	2.10E-43
Ccl9	656.87	12.66	6.82E-48	3.93E-45
A130071D04Rik	596.88	12.65	8.07E-33	2.55E-30
Il1b	396.85	12.60	1.40E-40	6.21E-38
Gm1966	423.74	12.60	1.50E-34	5.24E-32
Cd300lf	394.87	12.52	3.72E-35	1.36E-32
Blnk	1015.81	12.35	1.42E-70	2.05E-67
Nlrp1b	388.92	12.31	1.83E-46	1.03E-43
Crybb1	2631.46	12.20	2.47E-19	2.79E-17
Aif1	5410.21	12.20	2.00E-22	2.93E-20
Fcgr2b	339.37	12.16	2.28E-48	1.34E-45
Bcl2a1b	268.20	12.11	4.77E-41	2.20E-38

TRANSPARENT METHODS

Animals

All animal studies were performed according to the German animal care and ethics legislation and approved by the Committee on Research Animal Care, Regierungspräsidium Freiburg. Mice were maintained on a C57BL/6 background, under a 12-hour light and dark cycle and water and standard chow were provided ad libitum. The morning after a vaginal plug was detected was designated E0.5.

Brain dissociation

Brains from E14.5 embryos were acutely isolated and meninges were removed under a dissecting microscope. Four methods were trialed, with Liberase + DNase I performing the best according to cell survival, overall digestion as well as the retention of cell surface markers (Table S1). Each of the methodologies is detailed below.

Liberase and DNase I

E14.5 brains were transferred to 1.5 ml eppendorf tubes. To each tube, 475 µl of PBS containing 5 mM $MgCl_2$, 20 µl DNase I (final concentration 80 U/ml, New England Biolabs #M0303) and 5 µl Liberase TM (final concentration 0.13 WU/ml, Roche 05401119001) were added. Samples were incubated in a thermomixer (37°C, 800 rpm) for 40 minutes. Samples were gently triturated using a P1000 pipette at 20 minutes and again at the end of the 40 minutes. Samples were filtered through a 100 µm sieve and cells collected by centrifugation (250 g, room temperature, 4 minutes, swing bucket rotor) and resuspended in FACS buffer (PBS supplemented with 2% fetal calf serum).

The same methodology was used for the *Liberase only* test, with the exception that DNase I was excluded.

Pancreatin trypsin

Embryonic E14.5 brains were transferred to 500 µl dissociation solution containing 2.5% w/v pancreatin (Sigma P3292) and 0.5% w/v trypsin (Gibco 27250-018) in PBS. Tubes were incubated on ice for 30 minutes and flicked occasionally to ensure mixing. After 30 minutes, the dissociation solution was removed and samples incubated at 37°C for 5 minutes in a water bath. Next, 2% FCS / PBS was added and the brains mechanically dissociated using a P1000 pipette and passed through a 100 µm sieve prior to analysis.

Collagenase and dispase

Collagenase and Dispase were purchased from Roche (10269638001) and used according to the manufacturer's recommendations at a concentration of 1 mg/ml (w/v).

No enzyme

E14.5 brains were placed in FACS buffer and triturated ~10 times using a P1000 pipette. Samples were collected by centrifugation (250 g, room temperature, 4 minutes, swing bucket rotor) and tested for cell viability.

Flow cytometry

Dissociated E14.5 brains were washed once in PBS supplemented with 5 mM $MgCl_2$. Cells were passed through a 100 µm sieve, resuspended in 400 µl FACS buffer (PBS + 2% fetal calf serum) containing the PDGFRβ antibody (R+D, Table S2) and incubated on ice for 30 minutes. After two washes in FACS buffer at 4°C, cells were resuspended in 400 µl FACS buffer containing antibodies raised against CD11b, CD41, CD45, CD102 (ICAM2) and PECAM1 (CD31) as well as the Alexa488-conjugated anti-goat IgG (ThermoFisher A11055) at the indicated concentrations (Table S2). Zombie dye (Biolegend 423106, 1:200) was added to the mix as a viability marker. Cells were incubated on ice for 45 minutes, washed three times in ice cold FACS buffer, collected by centrifugation (250 g, 4°C) in a swinging bucket rotor and sorted on the FACS ARIA (BD Biosciences) using a 100 µm nozzle. For scRNA-seq analysis, cells were sorted into 384 well plates and processed according to the mCEL-Seq2 protocol (see below). For bulk populations, cells were collected in ice-cold FACS buffer, centrifuged (300 g, 4 minutes, 4°C) in a swing bucket rotor. Cell pellets were snap frozen and stored at -80°C. RNA from neural cells was isolated using the Qiagen mini kit (#74104), while RNA from mural cells, endothelial cells and microglia was extracted using the Qiagen miRNeasy Micro kit (#1071023). Libraries for RNA-seq of the neural population were prepared using the Illumina TruSeq library preparation kit. For mural cells, endothelial cells and microglia, cDNA was prepared from isolated RNA using the SMART-Seq v4 Ultra Low Input RNA Kit for Sequencing (Clontech 634891). Libraries were prepared using the Nextera XT DNA library preparation kit (Illumina FC-131-1096) and sequenced on the HiSeq 2500 instrument.

Single cell RNA sequencing

scRNA-seq was performed according to the mCEL-Seq2 protocol (Hashimshony et al., 2016; Herman et al., 2018). Single cells were index sorted into 384-well plates containing 240 nl of primer mix and 1.2 µl of mineral oil (Sigma-Aldrich). A FACS-ID for each individual well was assigned according to the gating strategy. The plates were centrifuged at 2200 g for 10 minutes at 4°C, snap-frozen in liquid nitrogen and stored at −80°C until processing. RNA was reverse transcribed using 160 nl of reverse transcription reaction mix and 2.2 µl of second strand reaction mix. cDNA from 96 cells was pooled together before clean up and *in vitro* transcription, generating 4 libraries from each 384-well plate. 0.8 µl of AMPure/RNAClean XP beads (Beckman Coulter) per 1 µl of sample were used during all the purification steps including the library clean up. Other steps were performed as described in the protocol (Herman et al., 2018). Libraries were sequenced on an Illumina HiSeq 3000 sequencing system (pair-end multiplexing run, high output mode) at a depth of ~200,000 reads per cell.

Bioinformatic analyses

Raw sequencing data for both single cell and bulk experiments were controlled for quality, trimmed and mapped to the reference mm10 genome using STAR (Dobin et al., 2013). The identity of reads

was identified through FeatureCount (Liao et al., 2014). For scRNA-seq analyses, the RNA expression matrices from each cell were merged and further analysed with the RaceID3 software package (Herman et al., 2018). Cells with less than 1500 unique transcripts were discarded, which left 625 cells for downstream analysis. For downstream analyses, ribosomal genes as well as the highly expressed ncRNA *Malat1* were removed. Cells were clustered using k-medoids and the clustering result was confirmed using the random forest algorithm within the RaceID3 package. Clusters from the scRNA-seq data were visualized through the two-dimension t-distributed stochastic neighbour embedding (tSNE) algorithm within the RaceID3 package. For both scRNA-seq and the bulk RNA-seq analyses, differentially expressed genes between the cell types or cell clusters were identified using DESeq2 (Love et al., 2014).

Inter-cellular ligand-receptor interaction networks were calculated based on the publically available database collated by Ramilowski and co-workers (Ramilowski et al., 2015). Mouse orthologs of human genes were obtained using the biomaRt R package (Durinck et al., 2005; Durinck et al., 2009). The connection between cell type and itself (autocrine) or another cell type (paracrine) was established if the receptor and its associated ligand were expressed in at least one of the four cell types. The ligand or receptor was considered expressed in each scRNA-seq cluster if the mean expression per cluster was higher than 0.7 normalised transcripts. In contrast, the ligand or receptor was considered expressed in the bulk RNA-seq dataset if the mean expression per cell type was higher than 10 FPKM, and the respective ligand or receptor expression in the given cell type was higher than 10% of maximal expression of the given ligand/receptor in any cell type. Directionality of a given interaction was determined by the cell type expressing the ligand. Colours in Figures 4B and S3B correspond to the cell type or cluster expressing the ligand, while the thickness of the lines is proportional to the number of interaction pairs between cell types. For visualization of interaction networks, the igraph (Csardi and Nepusz, 2006) and iTALK (Wang et al., 2019) R packages were utilized. The network of top 50 enriched interactions was generated with the additional criterion for the ligands to be enriched with an enrichment score > 0.5 in a cell type. For ranking the ligand-receptor interactions, the absolute expression (FPKM) of both the ligand and receptor in each interacting pair was taken into account. The enrichment score was calculated for each gene based on the normalized DESeq2 gene expression matrix; Mean expression of a gene across all cell types was subtracted from the expression levels of that gene in a particular cell type, and subsequently divided by the standard deviation. Thus, the enrichment score for a particular gene in a cell type represents the number of standard deviations from its mean expression across all analysed cell types.

Generation of the Brain Interactome Explorer website

The R code for generating circos plots from normalized expression values was wrapped in a shiny app using CRAN R package shiny version 1.3.2 (Chang et al., 2019) and R version 3.6. The resulting shiny app 'Brain Interactome Explorer' version 1.0.0 is served on shinyapps.io under https://mpi-ie.shinyapps.io/braininteractomeexplorer/. The R code underlying the app is available under https://github.com/maxplanck-ie/BrainInteractomeExplorer.

Immunofluorescence

Testing of vascular PECAM1, ICAM2, PDGFRβ and NG2 antibodies

Brains were isolated from E14.5 embryos, meninges removed and both cortices isolated. The dissected cortices were fixed in 4% paraformaldehyde at room temperature for 30 minutes. After brief washing, samples were stored in 100% methanol at 4°C. For staining, the cortices were washed in 50% methanol for 10 minutes, once in PBS for 10 minutes and twice in wash buffer (0.1% Triton X-100 in PBS) for 15 minutes each. Samples were blocked in 10% fetal calf serum (FCS) for 1 hour at room temperature and then incubated with primary antibodies raised against PDGFRβ and PECAM1 (CD31, (1:50), diluted in wash buffer supplemented with 10% FCS, overnight at 4°C. The cortices were subsequently processed through the wash buffer three times (10 minutes each) at 4°C and then three times at room temperature. The cortices were incubated with secondary antibodies (Donkey anti-goat Alexa488, A21206, 1:400; donkey anti-rabbit Alexa555, A31572, 1:400) diluted in FACS buffer and incubated at room temperature for 2 hours. The samples were then washed three times at 4°C for 10 minutes each and subsequently three times for 1 hour at room temperature. The cortices were subsequently stained with DAPI (1:1000 diluted in PBS) for 30 minutes at room temperature and washed three times for 10 minutes each. The stained cortices were flattened, mounted in Fluoromount and imaged using the LSM780 microscope from Zeiss using the 63x oil objective. Data were processed using the Zen Blue software (Zeiss).

Immunofluorescence analyses of overlap in protein localisation

Freshly dissected E14.5 brains were fixed in 4% paraformaldehyde at room temperature for 2 hours, washed with PBS and embedded in a 25% albumin; 6% gelatine solution. Embedded samples were fixed further in 4% paraformaldehyde overnight, washed and subsequently stored in PBS until sectioning. Approximately 100 μm thick sections were cut on a vibratome and slices were released in PBS. Each section was transferred to a well of a 24-well plate for subsequent staining. For stainings with antibodies against IBA1, APOE, LDLR, LRP1 and βIII-tubulin, sections were permeabilized and blocked in PBS containing 0.5% TritonX-100 and 0.25% gelatine overnight at 4°C and all antibody incubations were carried out in PBS supplemented with 0.25% gelatine. For stainings with antibodies raised against VTN, KDR, PECAM1, LAMA4 and ITGB1, sections were permeabilised and blocked in PBS containing 0.5% Triton X-100 and 10% FCS overnight at 4°C. All samples were incubated with the respective combinations of primary antibodies (1:200) overnight at 4°C, washed four times for 30 minutes each at room temperature, and incubated with the appropriate secondary antibodies (donkey anti-goat 594 (1:250, Life Technologies #A11058), donkey anti-rabbit 488 (1:250, Life Technologies #A11055), goat anti-mouse 594 (1:250, Life Technologies #A11032), donkey anti-rabbit 555 (1:250, Life Technologies #A31572), goat anti-mouse 488 (1:250, Life Technologies #A11001)) overnight at 4°C. After a further 4 washes in PBS, samples were stained with DAPI (1:250, 20 minutes, RT), mounted onto slides with Fluoromount and imaged using the Zeiss Airyscan LSM 880 microscope with the 25x oil objective. Data were processed using the Zen Blue software (Zeiss).

Co-immunoprecipitation assays

Cell lysates were prepared in HMG150 buffer (25 mM HEPES pH 7.6, 12.5 mM $MgCl_2$, 10% glycerol, 150 mM KCl, 0.5% Tween-20 and Protease Inhibitor Complete Mini (Roche #04693159001)) from wild type snap frozen E14.5 brains and 250 µg of protein were used for each IP. A mixture of Protein A/G beads (Sepharose 4 Fast Flow Protein G (GE Healthcare #17-0618-05) and Protein A (GE Healthcare #17-5280-02)) were blocked by incubating with 0.2 mg/ml BSA in HMG150 for 30 minutes at 4°C. After pre-clearing brain lysates with 100 µl of a Protein A/G beads-slurry, 5 µg of the anti-APOE antibody were added and samples incubated overnight at 4°C. IPs with rabbit IgG (Rb-IgG, Abcam #ab172730) were used as controls. Next, 100 µl of blocked Protein A/G beads-slurry were added, samples incubated at 4°C for 1 hour and washed 3 times in HMG150 for 10 minutes each. Immunoprecipitated protein was eluted in ROTI-load (Roth #K929.1), boiled briefly and run on a 4-12% gradient gel (NuPAGE, Life Technologies #NP0321Box). Following transfer onto 0.45 µm PVDF membranes (ImmobilonP Membrane, Millipore #IPVH00010), membranes were blocked in 5% skim milk, incubated overnight at 4°C with antibodies raised against APOE and LRP1 (1:1000). After washing, the membranes were incubated with the HRP-conjugated anti-rabbit or anti-goat antibodies (ECL anti-rabbit HRP, GE Healthcare #NA934 and ECL anti-goat HRP, Santa Cruz #sc2354) for 1 hour at room temperature, washed and developed on the Chemi Doc System (Biorad), using the Lumi-Light reagent (Lumi Light Plus Western Blotting Substrate, Roche #12015196001).

Data and software availability

The Brain Interactome Explorer is available at https://mpi-ie.shinyapps.io/braininteractomeexplorer/.

Raw sequencing data have been uploaded to GEO and are available under GSE133079.

SUPPLEMENTARY REFERENCES

Chang, W., Cheng, J., Allaire, J.J., Xie, Y., and McPherson, J. (2019). shiny: Web Application Framework for R. R package version 1.3.2. (https://CRAN.R-project.org/package=shiny).

Csardi, G., and Nepusz, T. (2006). The igraph software package for complex network research. InterJournal, Complex Systems *1695*.

Dobin, A., Davis, C.A., Schlesinger, F., Drenkow, J., Zaleski, C., Jha, S., Batut, P., Chaisson, M., and Gingeras, T.R. (2013). STAR: ultrafast universal RNA-seq aligner. Bioinformatics *29*, 15-21.

Durinck, S., Moreau, Y., Kasprzyk, A., Davis, S., De Moor, B., Brazma, A., and Huber, W. (2005). BioMart and Bioconductor: a powerful link between biological databases and microarray data analysis. Bioinformatics *21*, 3439-3440.

Durinck, S., Spellman, P.T., Birney, E., and Huber, W. (2009). Mapping identifiers for the integration of genomic datasets with the R/Bioconductor package biomaRt. Nat Protoc *4*, 1184-1191.

Hashimshony, T., Senderovich, N., Avital, G., Klochendler, A., de Leeuw, Y., Anavy, L., Gennert, D., Li, S., Livak, K.J., Rozenblatt-Rosen, O.*, et al.* (2016). CEL-Seq2: sensitive highly-multiplexed single-cell RNA-Seq. Genome Biol *17*, 77.

Herman, J.S., Sagar, and Grun, D. (2018). FateID infers cell fate bias in multipotent progenitors from single-cell RNA-seq data. Nat Methods *15*, 379-386.

Liao, Y., Smyth, G.K., and Shi, W. (2014). featureCounts: an efficient general purpose program for assigning sequence reads to genomic features. Bioinformatics *30*, 923-930.

Love, M.I., Huber, W., and Anders, S. (2014). Moderated estimation of fold change and dispersion for RNA-seq data with DESeq2. Genome Biol *15*, 550.

Ramilowski, J.A., Goldberg, T., Harshbarger, J., Kloppmann, E., Lizio, M., Satagopam, V.P., Itoh, M., Kawaji, H., Carninci, P., Rost, B.*, et al.* (2015). A draft network of ligand-receptor-mediated multicellular signalling in human. Nature communications *6*, 7866.

Rosenberg, A.B., Roco, C.M., Muscat, R.A., Kuchina, A., Sample, P., Yao, Z., Graybuck, L.T., Peeler, D.J., Mukherjee, S., Chen, W.*, et al.* (2018). Single-cell profiling of the developing mouse brain and spinal cord with split-pool barcoding. Science *360*, 176-182.

Sheikh, B.N., Dixon, M.P., Thomas, T., and Voss, A.K. (2012). Querkopf is a key marker of self-renewal and multipotency of adult neural stem cells. Journal of cell science *125*, 295-309.

Vanlandewijck, M., He, L., Mae, M.A., Andrae, J., Ando, K., Del Gaudio, F., Nahar, K., Lebouvier, T., Lavina, B., Gouveia, L.*, et al.* (2018). A molecular atlas of cell types and zonation in the brain vasculature. Nature *554*, 475-480.

Wang, Y., Wang, R., Zhang, S., Song, S., Jiang, C., Han, G., Wang, M., Ajani, J., Futreal, A., and Wang, L. (2019). iTALK: an R Package to Characterize and Illustrate Intercellular Communication. bioRxiv.

Zeisel, A., Hochgerner, H., Lonnerberg, P., Johnsson, A., Memic, F., van der Zwan, J., Haring, M., Braun, E., Borm, L.E., La Manno, G.*, et al.* (2018). Molecular Architecture of the Mouse Nervous System. Cell *174*, 999-1014 e1022.
